# Medicinal plants as alternative and adjunct antimicrobial agents in ear, nose, and throat (ENT) Infections

**DOI:** 10.1007/s10787-026-02251-0

**Published:** 2026-04-27

**Authors:** Serpil Demirci Kayıran, Talih Özdaş, Umay Merve Güven Bölgen, Tilbe Çevikelli, Merve Nur Şahan, Hazar Ertuğrul, Ali Işıldaklı, Sedef Özkurt, Yakup Acar, Ege Demirkıran

**Affiliations:** 1 Department of Pharmaceutical Botany, Faculty of Pharmacy, Çukurova University, Adana, Turkey; 2 Department of ENT, Adana City Training and Research Hospital, Health Science University, Adana, Turkey; 3 Department of Pharmaceutical Technology, Faculty of Pharmacy, Çukurova University, Adana, Turkey; 4 Department of Pharmaceutical Technology, Faculty of Pharmacy, Bahçeşehir University, Istanbul, Turkey

**Keywords:** Medicinal plant, Ear, Nose and throat diseases, Antimicrobial, Activity

## Abstract

Medicinal plants are widely used worldwide for ear, nose, and throat (ENT) disorders and have a long history of traditional application. This review aims to summarize current experimental and clinical evidence on medicinal plants used for ENT conditions such as otitis externa/media, tinnitus, vertigo, allergic rhinitis, pharyngitis, and laryngitis, and to support the identification of new plant species with antimicrobial potential against ENT pathogens. This review thoroughly summarizes recent developments from 2020 to 2025 and was conducted using electronic databases, including PubMed, Web of Science, Scopus, ScienceDirect, and Google Scholar, with predefined ENT and medicinal plants-related keywords. Frequently used species include *Lavandula angustifolia*,* Thymus vulgaris*,* Curcuma longa*,* Zingiber officinale*,* Origanum vulgare*,* Glycyrrhiza glabra*,* Mentha piperita*,* Matricaria chamomilla*, and *Syzygium aromaticum*, many of which show In vitro antibacterial, antifungal, or antiviral activity relevant to upper airway and oral/ENT infections. Evidence indicates that selected medicinal plants and their extracts or essential oils inhibit key ENT-related pathogens, including multidrug-resistant respiratory and pharyngeal bacteria. The compiled data, structured in comparative tables, highlight promising taxa and preparation types, and underscore gaps in clinical validation, standardization, and safety assessment. Overall, this review provides an evidence-based overview of ENT-related phytotherapy and a framework for future pharmacological and phytochemical studies aimed at developing novel plant-derived antimicrobials for ear, nose, and throat diseases.

## Introduction

**Fig. 1:**
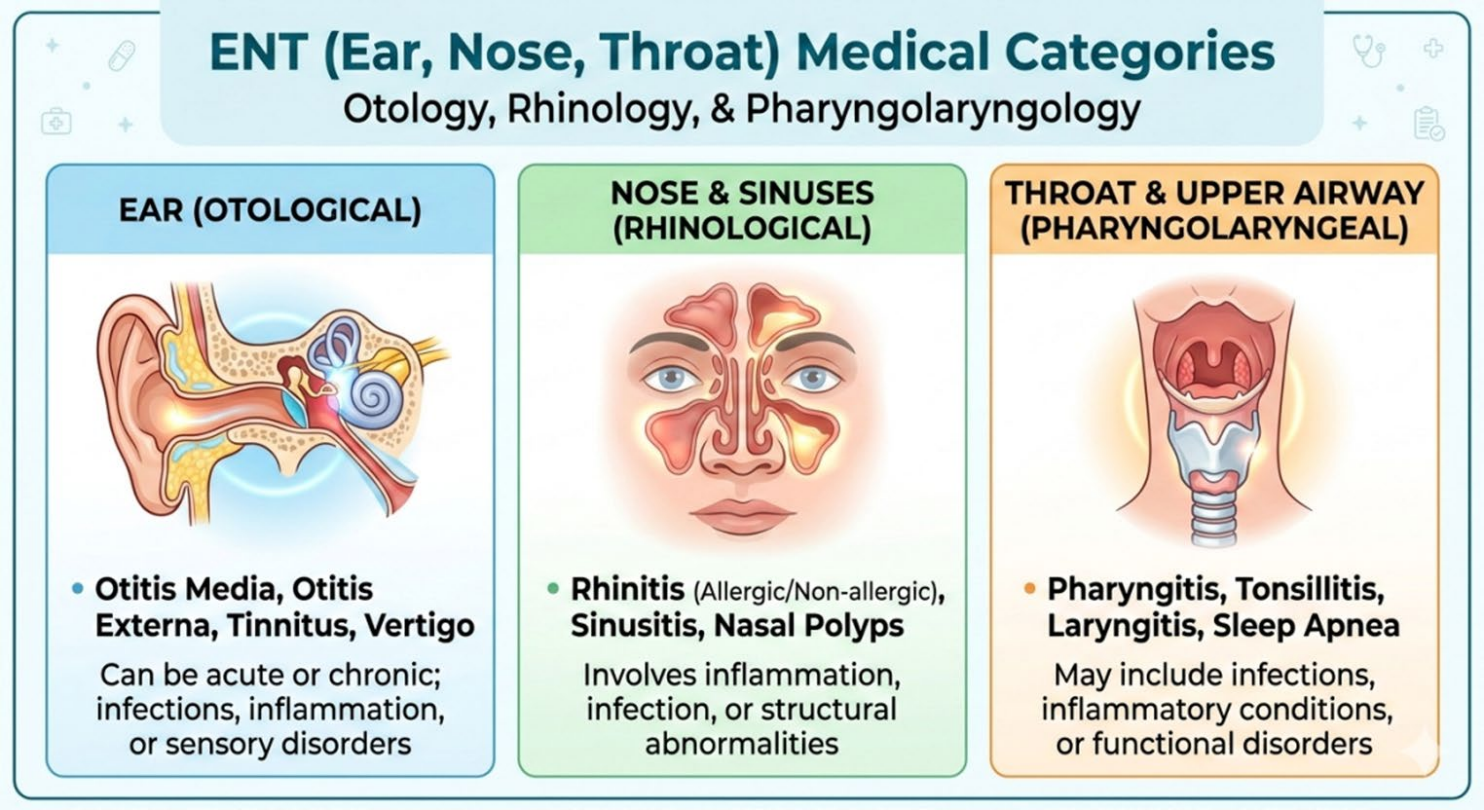
The classification of ENT diseases

Ear, nose and throat (ENT) diseases encompass a broad group of conditions affecting the upper respiratory tract and sensory organs, with both infectious and non-infectious origins. This system plays a role in vital sensory and functional processes such as hearing, balance, smell, taste and speech. Among ENT diseases such as otitis media, sinusitis, rhinitis, pharyngitis, tonsillitis, laryngitis, and allergic reactions are the most common (Fig. 1). In addition to bacterial or viral infections, environmental factors, allergens, cigarette smoke, air pollution, and immune system disorders are also play an important role in the etiology of these diseases. Clinical symptoms manifest as pain, congestion, secretion, hearing loss, hoarseness, and difficulty swallowing. In traditional medicine, various medicinal plants and natural products uses for many years to treat these diseases, and research into the scientific basis of these herbal treatments, alongside modern pharmacological approaches, is becoming increasingly important (Bhattacharyya et al. 2012; Brook 1996; Kumar and Clark 2020).

It is impossible to imagine that the human race could survive if there were no plants on Earth. Humankind has depended on plants since the beginning of time. Medicinal plants are common sources of medicine. There is solid evidence in ancient systems of medicine, such as Ayurveda, Unani and traditional Chinese medicine, that plant was used to treat diseases and repair and strengthen bodily systems. The innate desired purpose of the use of plants was to achieve a favorable interaction with body chemistry (Aslam and Ahmad 2016; Spinella 2001). The study of medicinal plant can be explained simply as “the study of the biologically active parts of plants and medicines that are used traditionally”. Consequently, the ethnopharmacological approach is based on a body of research spanning multiple disciplines, including botany, chemistry, and pharmacology. Field observations are included, as well as the utilization and bioactivities of folk remedies being described, the plant material being identified using botany, and phytochemical and pharmacological research being carried out. Many researchers have been interested in investigating the possible effects of indigenous remedies for a long time (Suntar 2020). Medicinal plants have employed by humanity since the early ages. The knowledge acquired through trial and error because of needs such as food, health and shelter has modified and developed until this time. This relationship between humankind and plants has given birth to the concept of ethnobotany (Kendir and Güvenç 2010). Medicinal plants have been used since the prehistoric, Mesopotamian, ancient Egyptian, Hittite, Greek, Roman, Seljuk and Ottoman periods (Özbek 2005). However, the tablets and inscriptions of the Sumerians and Egyptians contain information about medicinal plants (Dogan et al. 2017). The prescriptions used in the Mesopotamian records mention over 250 plants, including mandrake, henbane, hellebore, sweet flagroot, poppy, mustard, thyme, tragacanth, nutgall, mint, pomegranate peel, fennel, saffron and turpentine (Leonti and Verpoorte 2017). 600 plants identified in Ancient Greece, (Castro and Arias 2014) and 4000 plants in Arab and Persian culture (Burnes 2004). Undoubtedly, the modern Anatolian people have been influenced in using medicinal plants by the knowledge of civilisations from the Sumerians to the present day, as well as by the Central Asian and Islamic cultures from which they originated. The earliest written books on medicine by Chinese civilisation rulers mention medicinal herbs, introduce acupuncture therapy and explain the “yin-yang’’ theory (Qiao et al. 2022).

### Data collection and search strategy

This narrative review was conducted by researching for information on plants used in the treatment of ENT diseases. A range of electronic databases, including PubMed, Google Scholar, ScienceDirect, EBSCO, Web of Science, Scopus, ProQuest, HighWire Press, and the Cochrane Library, were systematically searched. Dating between 2020 and 2025 years, approximately 600 records were identified through database searching, of which 150 studies fulfilled the inclusion criteria and were included in the review. The studies included in the plant screening process were selected according to the following criteria: the research was conducted within an clinical or pharmacological framework focusing on plants used in the management of ENT disorders. The medicinal plants used for ENT-related conditions are presented in Tables, including their scientific name, family, chemical components, exract methods, treatments, study models, pharmacological effects, antimicribial activity and target species.

## Classification of ENT diseases

### Ear diseases

*Otitis externa (OE)* OE is an inflammation of the external auditory canal (Bradley et al. 2021). This condition is commonly referred to as swimmer’s ear and is frequently attributable to bacterial or fungal infections. Clinical manifestations may include otalgia, pruritus, otorrhea, and conductive hearing loss. Management typically involves aural toilet and the administration of topical antibiotic or antifungal drops.

*Otitis media (OM)* OM is characterized by inflammation of the middle ear and is particularly prevalent among preschool-aged children (Shirai and Preciado 2019). OM represents a spectrum of disease encompassing distinct clinical entities such as acute otitis media (AOM), otitis media with effusion (OME), and chronic suppurated otitis media (CSOM) (Cober and Johnson 2005). The most common bacterial pathogens associated with OM include *Streptococcus pneumoniae*,* Haemophilus influenzae*, and *Moraxella catarrhalis*. Diagnosis is based on clinical presentation, otoscopic evaluation of the tympanic membrane, and confirmation of Middle Ear Effusion. The American Academy of Pediatrics (AAP) guidelines emphasize the critical importance of accurate diagnosis in guiding treatment decisions (Shirai and Preciado 2019). Therapeutic approaches vary according to the type and severity of OM. In AOM, particularly in children under 2 years of age or in those presenting with severe symptoms, high-dose amoxicillin is the first-line treatment (Cober and Johnson 2005). Watchful waiting is recommended in children over 2 years of age with non-severe AOM as a strategy to reduce antibiotic use. Most cases of OME resolve spontaneously within three months, and the routine administration of antibiotics, decongestants, or antihistamines is not recommended during this period.

*Vertigo* Vertigo is the illusion of movement of oneself or the surrounding environment and arises from a disorder of the vestibular system (Stewart et al. 1999). Vertigo is classified into two major categories: peripheral and central. Peripheral vertigo results from pathologies involving the inner ear labyrinth or the vestibular nerve, whereas central vertigo originates from lesions in the brainstem, cerebellum, or cerebral hemispheres (Isaradisaikul et al. 2010). A detailed patient history and neuro-otological examination findings are of critical importance in the differential diagnosis. The most common causes of peripheral vertigo include benign paroxysmal positional vertigo (BPPV), Meniere’s disease, and vestibular neuritis (Omron 2019). BPPV is characterized by brief episodes of vertigo triggered by head movements and is diagnosed by the Dix–Hallpike maneuver. Meniere’s disease is defined by the tetrad of episodic vertigo, fluctuating sensorineural hearing loss, tinnitus, and aural fullness. Vestibular neuritis is typically a post-viral condition characterized by acute-onset, severe vertigo lasting hours to days (Herdman 2024). Therapeutic management depends on the underlying etiology and may include repositioning maneuvers, betahistine, diuretics, or antiemetics (Kentala et al. 1995).

*Tinnitus* Tinnitus is the perception of sound in the absence of an external acoustic stimulus. It is commonly described as simple sounds such as ringing, buzzing, or hissing and may be unilateral or bilateral. The prevalence of tinnitus in the adult population ranges from 10 to 15% and tends to increase with age. Risk factors for tinnitus include hearing loss, noise exposure, head trauma, anxiety, and depression (Baguley et al. 2013). The pathophysiology of tinnitus is complex and is generally thought to arise from pathological changes along the auditory pathways (Langguth et al. 2013). Diagnosis of tinnitus relies on medical history and symptom evaluation. Audiometric tests are performed to assess hearing function, and the severity and impact of tinnitus on quality of life are measured using questionnaires such as the Tinnitus Handicap Inventory. The primary goal of treatment is not the elimination of tinnitus but rather habituation and reduction of tinnitus-related distress. Management strategies include hearing aids for patients with hearing loss, sound therapy (e.g., white noise generators), and counseling. Cognitive-behavioral therapy (CBT) has the strongest evidence base for reducing tinnitus-related distress (Baguley et al. 2013).

### Nose diseases

*Acute rhinosinusitis (ARS)* ARS is an acute-onset inflammation of the nasal and paranasal sinus mucosa (Passali et al. 2015). It most commonly develops following a viral upper respiratory tract infection, with symptoms including nasal obstruction, rhinorrhea, facial pain/pressure, and hyposmia. Differentiating between viral and bacterial ARS is of clinical importance for guiding appropriate management and preventing unnecessary antibiotic use. Treatment is primarily symptomatic and typically includes nasal irrigation, decongestants, and intranasal corticosteroids (Vardouniotis et al. 2025).

*Allergic rhinitis (AR)* AR is an immunoglobulin E (IgE)-mediated inflammatory response of the nasal mucosa following exposure to inhaled allergens (Seidman et al. 2015). This highly prevalent condition affects millions worldwide and is characterized by symptoms such as sneezing, rhinorrhea, nasal pruritus, and nasal obstruction (Greiner et al. 2011). Traditionally, AR has been classified as seasonal or perennial (Okubo et al. 2020). However, the allergic rhinitis and its Impact on Asthma (ARIA) guidelines propose a more functional classification based on symptom duration (intermittent or persistent) and impact on quality of life (mild or moderate–severe) (Greiner et al. 2011). The pathophysiology involves both early- and late-phase reactions (Okubo et al. 2020). In the early phase, allergen cross-linking of IgE antibodies bound to mast cells within the nasal mucosa induces the release of chemical mediators such as histamine, triggering acute symptoms including sneezing and watery rhinorrhea (Seidman et al. 2015).

Pharmacotherapy constitutes the cornerstone of management. Intranasal corticosteroids are regarded as the most effective monotherapy, targeting both early- and late-phase reactions and providing relief across the full spectrum of nasal symptoms (Seidman et al. 2015). Oral second-generation antihistamines are recommended particularly for patients with predominant sneezing and pruritus. Intranasal antihistamines represent an additional therapeutic option (Brozek et al. 2017). In patients with inadequate symptom control on monotherapy, a combination of intranasal corticosteroids with either oral or intranasal antihistamines may be considered. Leukotriene receptor antagonists (LTRAs) are another option, although they are generally not recommended as first-line therapy. Allergen immunotherapy (sublingual or subcutaneous) remains the only treatment modality capable of modifying the natural course of the disease and should be considered in patients with insufficient response to pharmacotherapy (Greiner et al. 2011).

### Throat diseases

*Acute tonsillitis (AT)* AT is an inflammatory condition, typically of infectious origin, affecting the palatine tonsils (Sidell and Shapiro 2012). Although most commonly observed in school-aged children, it can occur at any age. Viral agents are the predominant etiology, including adenoviruses, Epstein–Barr virus (EBV), influenza, and parainfluenza viruses (Windfuhr et al. 2016). The most significant and frequently encountered bacterial cause is group A beta hemolytic streptococcus (GABHS), specifically *Streptococcus pyogenes*.

Clinical presentation varies according to etiology but generally includes sore throat, fever, dysphagia, odynophagia, and tender cervical lymphadenopathy. Clinical scoring systems, such as the Centor or McIsaac criteria, are utilized to estimate the likelihood of GABHS infection (Sotirović 2022). When scores indicate a high probability of bacterial infection (e.g., Centor score ≥ 3), confirmatory testing via rapid antigen detection or throat culture is recommended. Management is etiology-directed: supportive care including rest, hydration, and analgesics is sufficient for viral tonsillitis. For confirmed GABHS tonsillitis, the treatment of choice is a 7–10 day course of penicillin to prevent nonsuppurative complications such as rheumatic fever. In patients with penicillin allergy or treatment failure, alternative antibiotics such as cephalosporins or macrolides may be employed (Windfuhr et al. 2016).

*Acute pharyngitis (AP)* AP is an inflammatory condition of the pharynx and/or tonsils, commonly observed in both children and adults. It accounts for approximately 1–2% of all outpatient visits in the United States. The etiology is diverse, with viral causes predominating, followed by bacterial agents. Viral pharyngitis typically presents with symptoms such as cough, rhinorrhea, and conjunctivitis, which are less common in bacterial pharyngitis (Caldwell et al. 2024).

The most common bacterial pathogen is group A *Streptococcus* (GAS), responsible for 5–15% of adult cases and 20–30% of pediatric cases. GAS pharyngitis manifests with sore throat, fever, headache, and chills. Clinical decision rules, such as the McIsaac score, are employed to distinguish GAS pharyngitis from viral causes. In cases with a high clinical score, diagnosis is confirmed using rapid antigen detection tests and/or throat cultures. Acute pharyngitis associated with the Omicron variant of COVID-19 may present with distinctive findings, including marked mucosal congestion, without edema or tonsillar enlargement (Zhou et al. 2025) (Table 1).

Management is etiology-directed. Viral pharyngitis is treated symptomatically, whereas GAS pharyngitis requires antibiotic therapy typically penicillin or amoxicillinto prevent post-infectious sequelae such as acute rheumatic fever. Other bacterial pathogens, including groups C and G *Streptococcus*,* Fusobacterium necrophorum*, and *Arcanobacterium haemolyticum*, may alter the treatment regimen when detected.

**Table 1:** Acute pharyngitis etiology and key clinical features

Feature / pathogen	Children (%)	Adults (%)	Common Symptoms
Viral Pharyngitis	70–80	85–90	Cough, rhinorrhea, conjunctivitis
GAS (Group A Streptococcus)	20–30	5–15	Sore throat, fever, headache, chills
Other bacterial (Groups C/G, Fusobacterium, Arcanobacterium)	< 5	< 5	Variable, may require tailored antibiotics

*Upper respiratory tract infections (URTIs)* URTIs are a group of contagious illnesses, usually of viral origin, affecting the upper airways, including the nose, sinuses, pharynx, larynx, and trachea (Tobin et al. 2025). URTIs are highly prevalent in the community, and multiple episodes per year are considered normal, particularly in childhood (Allan and Arroll 2014). Most cases resolve spontaneously, although bacterial superinfections may occasionally occur (Allan and Arroll 2014; Chow et al. 2012). URTIs encompass infections involving various anatomical sites, including nasopharyngitis (common cold), pharyngitis, laryngitis, sinusitis, and occasionally otitis (Tobin et al. 2025). Acute URTI is typically diagnosed when symptoms resolve spontaneously within 10 days (Allan and Arroll 2014; Jaume et al. 2020).

Viral agents account for approximately 90% of URTIs and include rhinoviruses (most frequent), seasonal coronaviruses, adenoviruses, respiratory syncytial virus (RSV), influenza A and B, parainfluenza viruses, and enteroviruses (Allan and Arroll 2014; Tobin et al. 2025). Bacterial pathogens are rarely primary causes and more commonly occur as superinfections; the main agents include *Streptococcus pneumoniae*,* Haemophilus influenzae*,* Moraxella catarrhalis*, and *Streptococcus pyogenes* (Chow et al. 2012; Harmes et al. 2013; Linder et al. 2025). Clinically, patients present with rhinorrhea, nasal congestion, sore throat, low-grade fever, sneezing, cough, headache, hoarseness, myalgia, and malaise (Allan and Arroll 2014; Linder et al. 2025). Symptoms typically peak within a few days and resolve within 7–10 days (Allan and Arroll 2014). Viral infections generally produce more diffuse and milder symptoms, whereas bacterial superinfections are more localized and severe (Chow et al. 2012). Physical examination may reveal nasal mucosal edema and hyperemia, pharyngeal erythema, mild tonsillar swelling, and cervical lymphadenopathy; auscultation usually reveals no adventitious lung sounds. Productive cough may warrant consideration of bronchitis (Tobin et al. 2025).

Diagnosis is primarily clinical, and laboratory tests are generally unnecessary (Allan and Arroll 2014; DeGeorge et al. 2019). Further evaluation is warranted if symptoms persist beyond 10 days, there is high fever or systemic toxicity, or if unilateral facial/dental pain is present (Chow et al. 2012; Jaume et al. 2020). Rapid antigen detection or throat culture is indicated when streptococcal pharyngitis must be differentiated (Ashurst and Edgerley-Gibb 2018; Linder et al. 2025). Imaging should be reserved for complicated or atypical cases (e.g., suspected sinusitis, otitis, pneumonia) (Chow et al. 2012; Sur and Plesa 2022). First-line management is supportive and symptomatic. Paracetamol or ibuprofen is used for fever and pain control (DeGeorge et al. 2019). Nasal decongestants may be recommended short-term (3–5 days), with prolonged use risking rebound congestion (Craig 2025; FDA 2021; Norfleet 2025). Saline nasal irrigation can facilitate clearance of secretions. Adequate hydration, rest, throat lozenges and sprays, and humidified steam are effective in symptom relief (DeGeorge et al. 2019). Antibiotic therapy is unnecessary in most URTIs because viral infections do not respond to antibiotics (Sur and Plesa 2022; Yoon et al. 2017). Antibiotics are reserved for secondary bacterial sinusitis, streptococcal pharyngitis, otitis media, or severe superinfections in patients with chronic conditions such as COPD (Chow et al. 2012; Linder et al. 2025; Sur and Plesa 2022). In these cases, amoxicillin or amoxicillin–clavulanate is first-line; macrolides (azithromycin, clarithromycin) are alternatives in patients with penicillin allergy (Chow et al. 2012; Sur and Plesa 2022).

## General properties of medicinal plants

### Chemical constituents and pharmacological activities of medicinal plants

Medicinal plants are an important source of biologically active secondary metabolites with pharmaceutical potential (Farnsworth 1988; Ncube et al. 2008). Approximately 50,000 higher plant species have been used in traditional medicine, yielding diverse classes of natural compounds including alkaloids, flavonoids, terpenoids, tannins, steroids, and carbohydrates. More than 4,000 phytochemicals have been identified, with around 150 extensively studied for their biological activities. These compounds serve as therapeutic agents, lead molecules, or intermediates for drug development (Rates 2001).

**Fig. 2:**
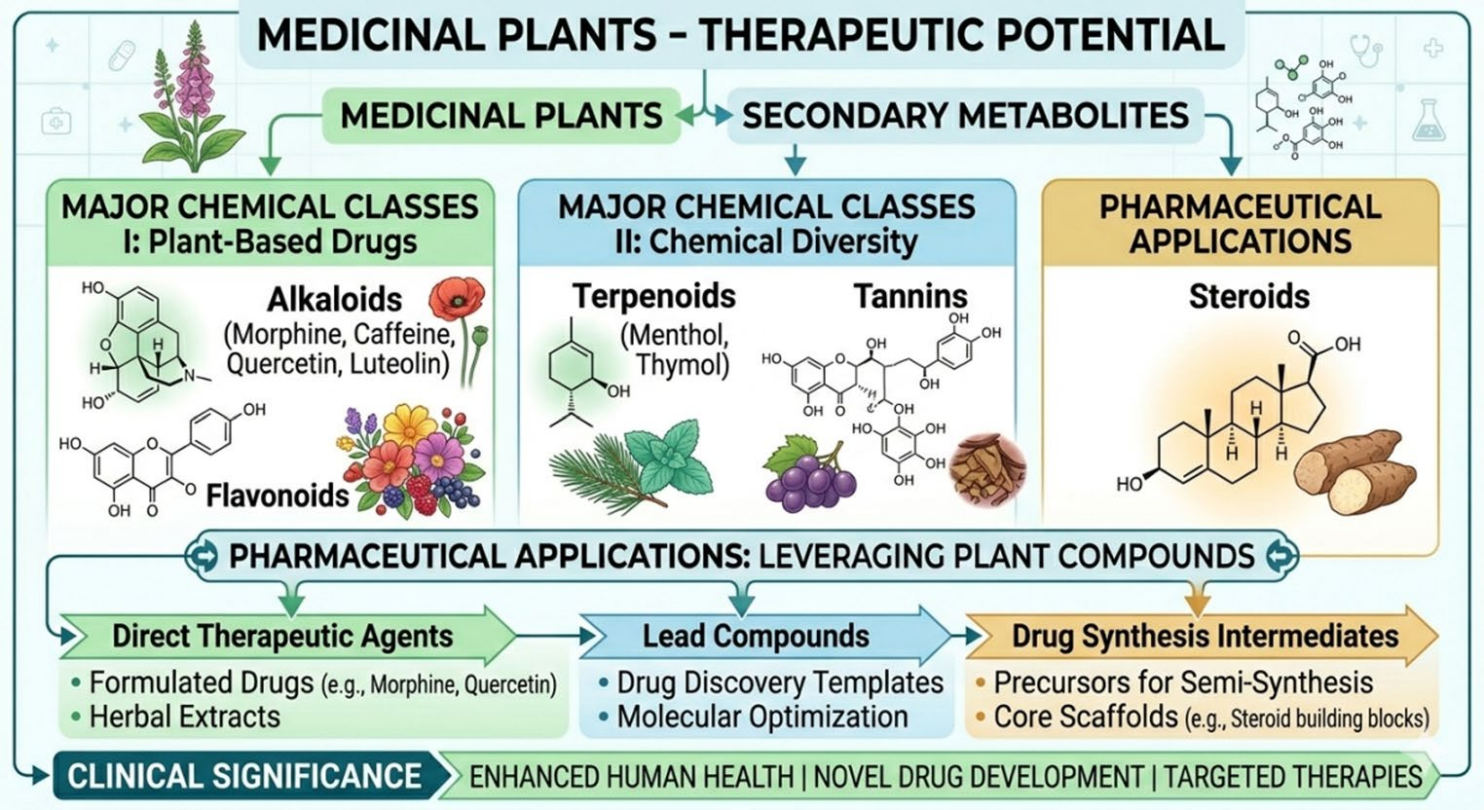
Pharmaceutical importance of plant-derived secondary metabolites

Medicinal plants display diverse pharmacological activities (e.g., antimicrobial, antioxidant, antiinflamatuary, cytotoxic, adaptive, stimulatory, and sedative properties). They are used as cholagogic, hypotensive, capillary-enforcing, antiulcer, anticholinesterase, anticancer, spasmolytic, analgesic, and analeptic medications (Yansheng Wang et al. 2022). An advantage of medicinal plants is that they provide patients with a complex of natural compounds, have smoother action and are better tolerated than synthetic drugs, and produce few allergic reactions. They do not accumulate and therefore can be administered for a long time. Medicinal plants and phytopreparations are used for therapy and prevention of various human diseases, including cardiovascular, gastrointestinal, nervous, and skin diseases, and even malignancies (Akram et al. 2014; Dahanukar et al. 2000) (Fig. 2).

## Medicinal plants used in ENT diseases

**Fig. 3:**
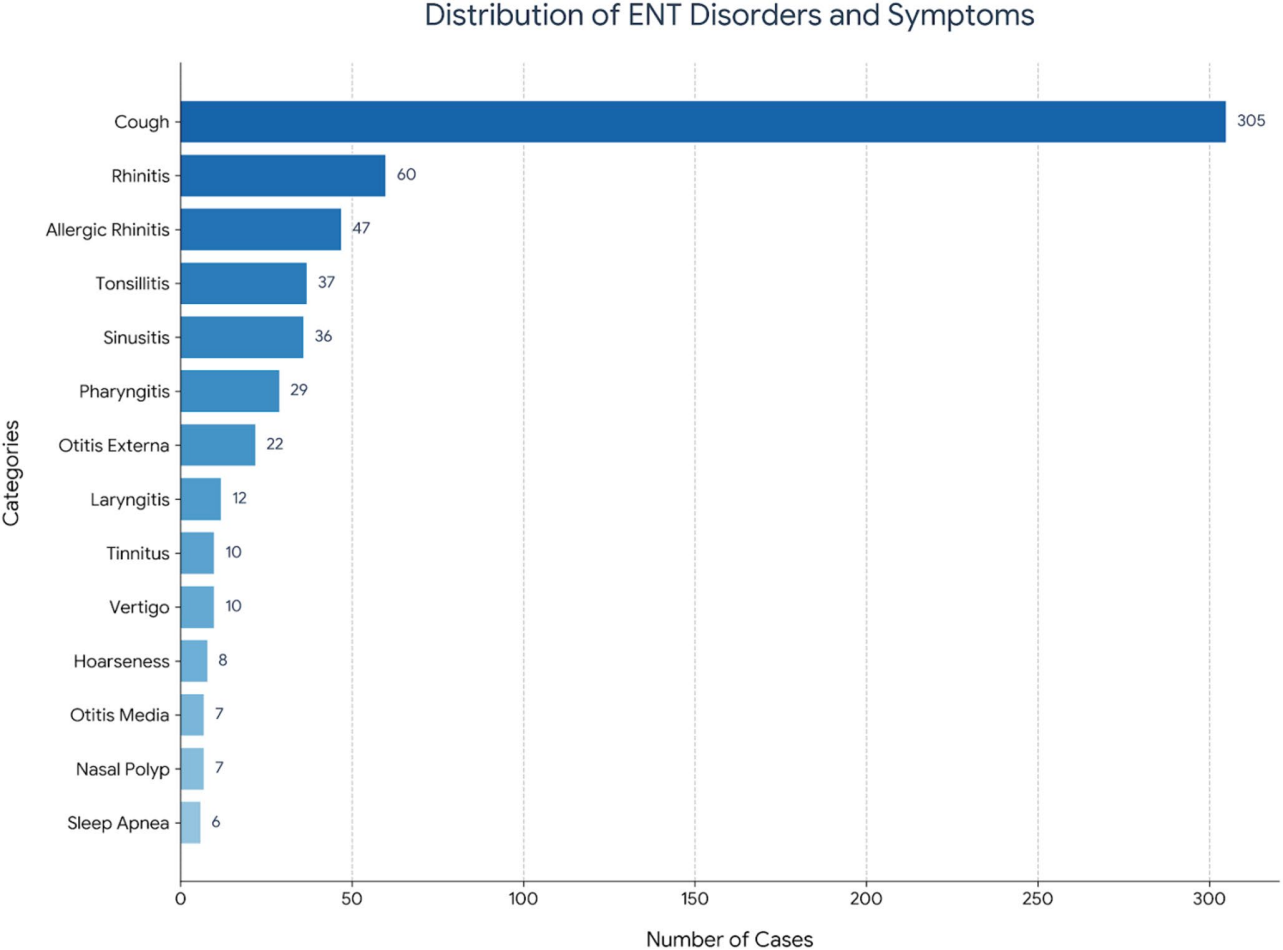
Bibliometric distribution of medicinal plant-based studies in ENT disorders published between 2020 and 2025. Cough-related studies are the majority (51.2%), followed by rhinitis and allergic rhinitis. Less-studied conditions include sleep apnea, nasal polyps, and otitis media, indicating potential gaps for future research

Between 2020 and 2025, a total of 596 studies investigated the use of medicinal plants in ENT disorders. The majority of these studies focused on cough (305 studies, 51.2%), followed by rhinitis (60 studies, 10.1%) and allergic rhinitis (47 studies, 7.9%). Other conditions such as tonsillitis (37 studies, 6.2%), sinusitis (36 studies, 6.0%), and pharyngitis (29 studies, 4.9%) were also investigated, whereas less frequently studied disorders included otitis externa (22 studies, 3.7%), laryngitis (12 studies, 2.0%), tinnitus (10 studies, 1.7%), vertigo (10 studies, 1.7%), hoarseness (8 studies, 1.3%), otitis media (7 studies, 1.2%), nasal polyps (7 studies, 1.2%), and sleep apnea (6 studies, 1.0%) (Clarivate Analytics 2025). This bibliometric analysis highlights the predominant research focus on respiratory symptoms, particularly cough, while other ENT conditions remain relatively underexplored, suggesting potential areas for future investigation (Fig. 3).

### Otitis externa

*Coptis chinensis* Berberine extract obtained from the root of *C. chinensis* was applied In vitro at various concentrations, and its antibacterial activity was assessed. The study tested on 12 bacterial strains and demonstrated that berberine significantly inhibited bacterial growth, particularly by disrupting *P. aeruginosa* biofilms and compromising cell membrane integrity. These findings suggest that berberine may serve as a potential phytotherapeutic agent in the treatment of otitis externa, offering an alternative approach in the context of rising antibiotic resistance (Olson et al. 2023).

*Zingiber officinale* A methanolic extract from the rhizomes of *Z. officinale* (ginger) was applied to various bacterial strains. Twenty bacterial isolates were tested, and the ginger extract demonstrated significant inhibition, particularly against *S. aureus* and *E. coli* strains. The effect was attributed to phenolic compounds such as gingerol and shogaol, which disrupt bacterial cell wall integrity and inhibit protein synthesis. These findings suggest that ginger extract may serve as a supportive phytotherapeutic option in the management of otitis externa (Al-Attraqchi et al. 2019).

*Thymus vulgaris* The essential oil of *T. vulgaris* (thyme) significantly inhibited the growth of *S. aureus* and *P. aeruginosa* by damaging bacterial cell membranes and suppressing inflammatory cytokine release. Consequently, essential oil of *T. vulgaris* demonstrated potential as a natural antimicrobial and anti-inflammatory agent for otitis externa treatment (Boukhatem et al. 2020).

*Matricaria chamomilla* The extract of *M. chamomilla* (chamomile) disrupted bacterial cell membranes and inhibited metabolic activity, demonstrating significant antimicrobial activity against *S. aureus*,* P. aeruginosa*, and *S. pneumoniae*. These findings suggest that chamomile exerts antibacterial effects that may support its topical use in otitis externa (Farsi et al. 2024).

*Sempervivum tectorum* The extracts of *S. tectorum* (houseleek) weretested In vitro against bacterial isolates from canine on otitis externa by Dégi et al. (2023). The extract, rich in phenolic and flavonoid compounds, demonstrated antibacterial activity by inhibiting the growth of pathogenic bacteria commonly isolated from infected dog-ears. The results indicate that *S. tectorum* could be a potential topical herbal alternative for managing otitis externa in veterinary practice.

*Syzygium aromaticum* Freitas et al. (2025) evaluated that the essential oil of *S. aromaticum* (clove) inhibited bacterial growth by disrupting the cell wall and interfering with energy metabolism. These results highlight the antibacterial potential of essential oil of *S. aromaticum* as a natural adjunct for treating resistant bacterial infections in otitis externa.

*Trachyspermum ammi* The essential oil of *T. ammi* (ajwain), rich in thymol, was incorporated into a chitosan-based hydrogel and tested In vitro against canine otitis externa pathogens. The chitosan–EO combination exhibited strong antibacterial activity against *Staphylococcus* spp. and *P. aeruginosa*, enhanced by the sustained-release property of the hydrogel. The study demonstrates the potential of essential oil based hydrogel formulations of *T. ammi* for topical management of otitis externa.

**Fig. 4:**
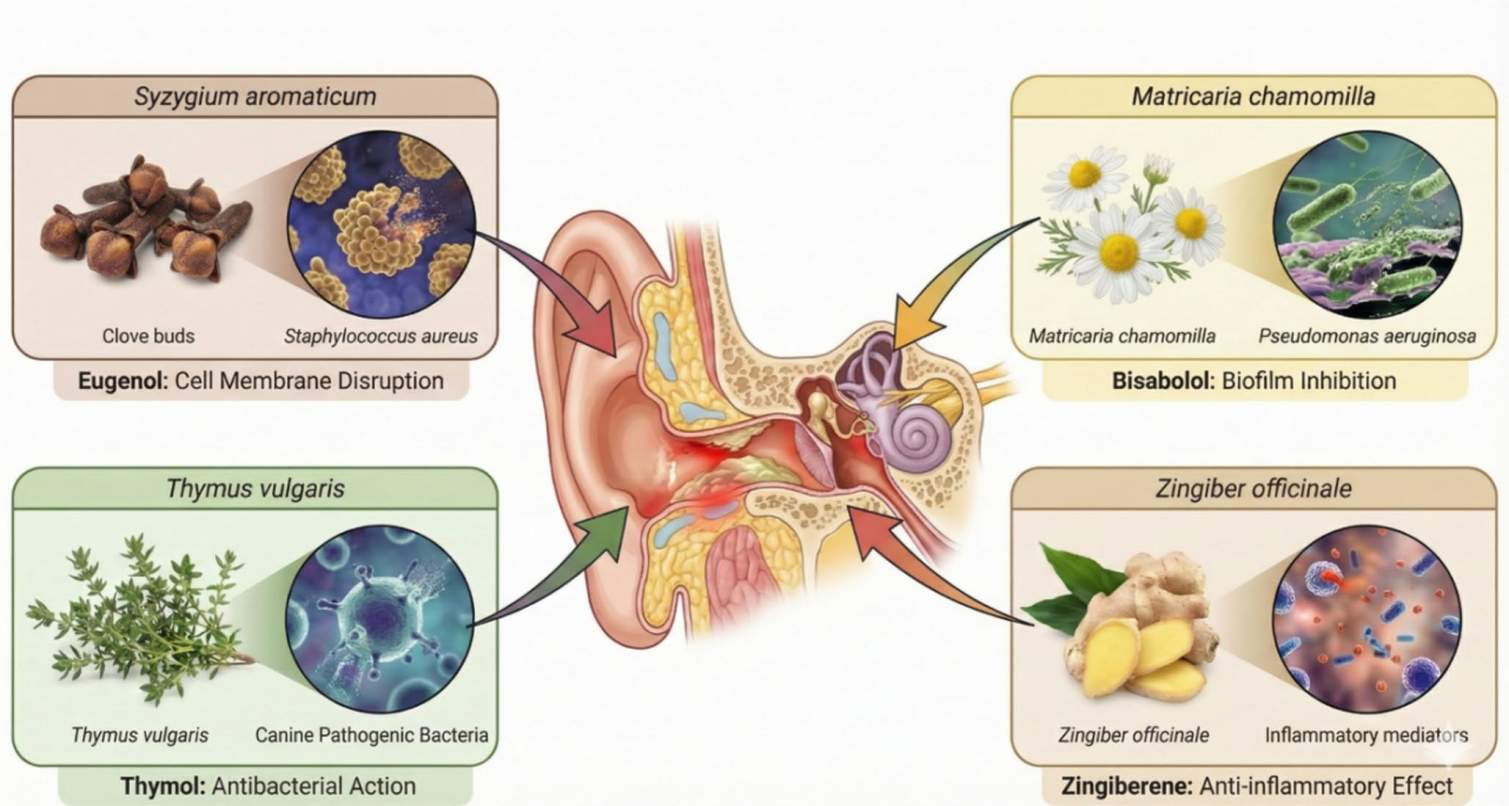
Comprehensive medicinal plant against otitis externa and their pharmacological activity

*Lavandula angustifolia* The essential oil and in combination with gentamicin of *L. angustifolia* demonstrated significant antibiofilm and antibacterial effects by disrupting biofilm structure and reducing bacterial viability. The findings suggest that essential oil of *L. angustifolia* may serve as a supportive natural agent for combating biofilm-associated otitis externa infections (Mourão et al. 2024) (Fig. 4; Table 2).

**Table 2:** Medicinal plants used for Otitis externa

Plant species	Chemical components	Exracts methods	Treatment	Model	Pharmacological effect	Target species	References
*Coptis chinensis*	Berberin, palmatin	Obtained in powder form from rhizome	Otitis externa	In vitro	Antibacterial effect; inhibits the growth of pathogens	*Staphylococcus spp.*,* Pseudomonas spp.*	Olson et al. (2023)
*Zingiber officinale*	Zingiberene, β-sesquiphellandrene	Essential oil distillation	Otitis externa	In vivo	Anti-inflammatory effects; reduces the expression of inflammatory cytokines	*Staphylococcus aureus*,* Pseudomonas aeruginosa*	Al-Attraqchi et al. (2019)
*Thymus vulgaris*	Carvacrol, thymol	Essential oil distillation	otitis externa	In vitro, In vivo	Antibacterial and anti-inflammatory effects; inhibits the growth of some pathogens	*Staphylococcus aureus*,* Pseudomonas aeruginosa*	Boukhatem et al. (2020)
*Matricaria chamomilla*	*α*-bisabolol, chamazulen, apigenin	Ready-made commercial products	Otitis externa	In vitro	Antimicrobial effect by disrupting bacterial cell membrane and inhibiting metabolism	*Staphylococcus aureus*,* Pseudomonas aeruginosa*,* Streptococcus pneumoniae*	Farsi et al. (2024)
*Sempervivum tectorum*	NR (phenolics/flavonoids commonly reported)	Plant extract (method NR)	Extract tested against OE pathogens from dogs	In vitro	Antibacterial	*Pathogenic bacteria from canine OE*	Dégi et al. (2023)
*Syzygium aromaticum*	Eugenol, eugenyl acetate, β-caryophyllene	Steam distillation (essential oil)	Topical EO; tested vs. MRSP from canine OE	In vitro (clinical isolates from dogs)	Antibacterial (growth inhibition of oxacillin-resistant S. pseudintermedius)	*Staphylococcus pseudintermedius (canine)*	(Freitas et al. 2025)
*Trachyspermum ammi*	Thymol-rich EO constituents	Steam distillation (EO) embedded in chitosan hydrogel	Chitosan hydrogel + T. ammi EO for canine OE pathogens	In vitro (dog isolates)	Antibacterial; enhanced by hydrogel delivery	*Bacterial pathogens from dogs (e.g.*,* Staphylococcus spp.*,* P. aeruginosa)*	Niaraki et al. (2024)
*Lavandula angustifolia*	Linalool, linalyl acetate (typical of lavender EO)	Steam distillation (EO) used alone and in an otological gel	EO/gel ± gentamicin against P. aeruginosa biofilm	In vitro	Antibiofilm/antibacterial vs. P. aeruginosa biofilm	*Pseudomonas aeruginosa (canine isolates)*	Mourão et al. (2024)

### Otitis media

*Aloe barbadensis* The ethanol extract obtained from the leaves of *A.barbadensis* showed an antibiofilm effect on pathogens such as *S. aureus* and *P. aeruginosa*. Mechanistically, it was found to be effective against the resistant form of infection by suppressing bacterial biofilm formation. Consequently, *A. barbadensis* extract stands out as a potential phytotherapeutic agent in the treatment of otitis media (Jotic et al. 2024).

*Zingiber officinale* The antibiofilm effect of ethanol extract obtained from the *Z. officinale* (ginger) was observed on *S. aureus* and *P. aeruginosa* strains. The proposed mechanism involves the interaction of gingerol and related phenolic compounds with the bacterial cell wall, leading to inhibition of bacterial adhesion and subsequent biofilm formation. With these effects, ginger extract has the potential to serve as an alternative phytotherapeutic agent in the treatment of otitis media (Jotic et al. 2024).

*Curcuma longa* In the study, an antibiofilm effect of *C. longa* was observed on *S. aureus* and *P. aeruginosa* strains. Curcumin has been shown to attenuate inflammation in host tissues, in part by disrupting bacterial biofilm structure due to its antioxidant and anti-inflammatory properties. Consequently, *C. longa* extract shows strong potential as a herbal agent in the treatment of otitis media (Jotic et al. 2024).

*Acacia Arabica* In the study conducted an antibiofilm effect of *A. arabica* was observed on *S. aureus* and *P. aeruginosa In vitro* model. The proposed mechanism involves the interaction of flavonoids and tannins with the bacterial cell wall, leading to inhibition of bacterial adhesion and subsequent biofilm formation. Consequently, *A. arabica* is considered a potential phytotherapeutic agent in the treatment of otitis media (Jotic et al. 2024).

*Ficus exasperate* In the study, it exhibited antibacterial effects of *F. exasperate* on *Klebsiella pneumoniae*,* P. aeruginosa*, and *S. aureus* strains. The mechanism was reported to be that phenolic compounds cause oxidative stress, disrupting the bacterial cell membrane and thus reducing the pathogen load. These results indicate that *F. exasperata* may be a strong phytotherapeutic candidate for the treatment of otitis media (Jotic et al. 2024).

*Securinega virosa* In the study, a significant antibacterial effect of *S. virosa* was detected against *K. pneumoniae*,* P. aeruginosa*,* S. aureus*, and *S. pneumoniae* bacteria. It was stated that the bioactive components of *S. virosa* contribute to infection control by inhibiting bacterial growth (Jotic et al. 2024).

*Tamarindus indica* In vitro tests reported antibacterial activity of *T. indica* against *K. pneumoniae*,* P. aeruginosa*,* S. aureus*, and *S. pneumoniae* strains. It was concluded that the phytochemical content of T. indica could be beneficial in the treatment of otitis media by inhibiting the growth of pathogenic microorganisms (Jotic et al. 2024).

*Thymus vulgaris* In vitro studies have determined that it exhibits a synergistic effect of *T. vulgaris* against multi-drug resistant fungal isolates. Thymol and carvacrol are known to exhibit antimicrobial activity by disrupting the cell membrane. These results indicate that *T. vulgaris* may be an effective complementary treatment for resistant infections (Ghaly et al. 2020).

*Syzygium aromaticum* In the study revealed a synergistic effect of *S. aromaticum* on multi-drug resistant fungal strains. It was noted that eugenol’s potent antimicrobial properties reduced the microbial load in the infected area. *S. aromaticum* has been identified as a promising natural agent for otitis media treatment due to these properties (Ghaly et al. 2020).

*Calendula officinalis* Mădălina Petran et al. (2024), conducted in their ethnobotanical study with 326 mothers with children in southern Romania. It was investigated the medicinal plants used for childhood illnesses and reported that *C. officinalis* was particularly widely preferred for skin diseases and mucosal irritations. Randomised clinical trials were examined the analgesic and symptom-relieving activities of multi-component herbal ear drops containing *C. officinalis* in children with acute otitis media. The study was showed that these preparations can reduce pain and discomfort to a degree similar to placebo or standard treatment, and in some studies, they have provided superior results. However, as these preparations typically contain a variety of plant extracts, including *Allium sativum* and *Hypericum perforatum*, it is not possible to attribute the observed effect solely to *C. officinalis*. Therefore, clinical studies that are well designed and use monocomponent preparations are needed to establish whether *C. officinalis* is effective and safe in the treatment of otitis media (Petran et al. 2024).

*Bryophyllum pinnatum* The antibacterial effect of aqueous and ethanolic leaf extracts of *B. pinnatum* was investigated using the agar well diffusion method on *S. aureus* and *P. aeruginosa* strains isolated from ear swabs of patients diagnosed with otitis media in Nigeria. Zone diameters and minimum inhibitory/minimum bactericidal concentration (MIC/MBC) values were measured. The study reported that both extracts produced significant growth inhibition against these pathogens, particularly at higher concentrations, with the ethanolic extract showing more pronounced antibacterial activity against certain strains. These results suggest that *B. pinnatum* may be a phytotherapeutic agent that could complement conventional antibiotics in the treatment of bacterial infections associated with otitis media (Sani 2020).

*Echinacea purpurea* In a clinical nutrition study, conducted by Pham et al. (2025), the effects of *E. purpurea* were evaluated on upper respiratory tract infections and otitis media. The study was conducted on human models using liquid extracts and tinctures have reported immunostimulatory, anti-inflammatory and antimicrobial effects. It has been suggested that *E. purpurea* is effective in preventing or alleviating attacks of otitis media by enhancing the immune response (Taufani and Ha 2025).

**Fig. 5:**
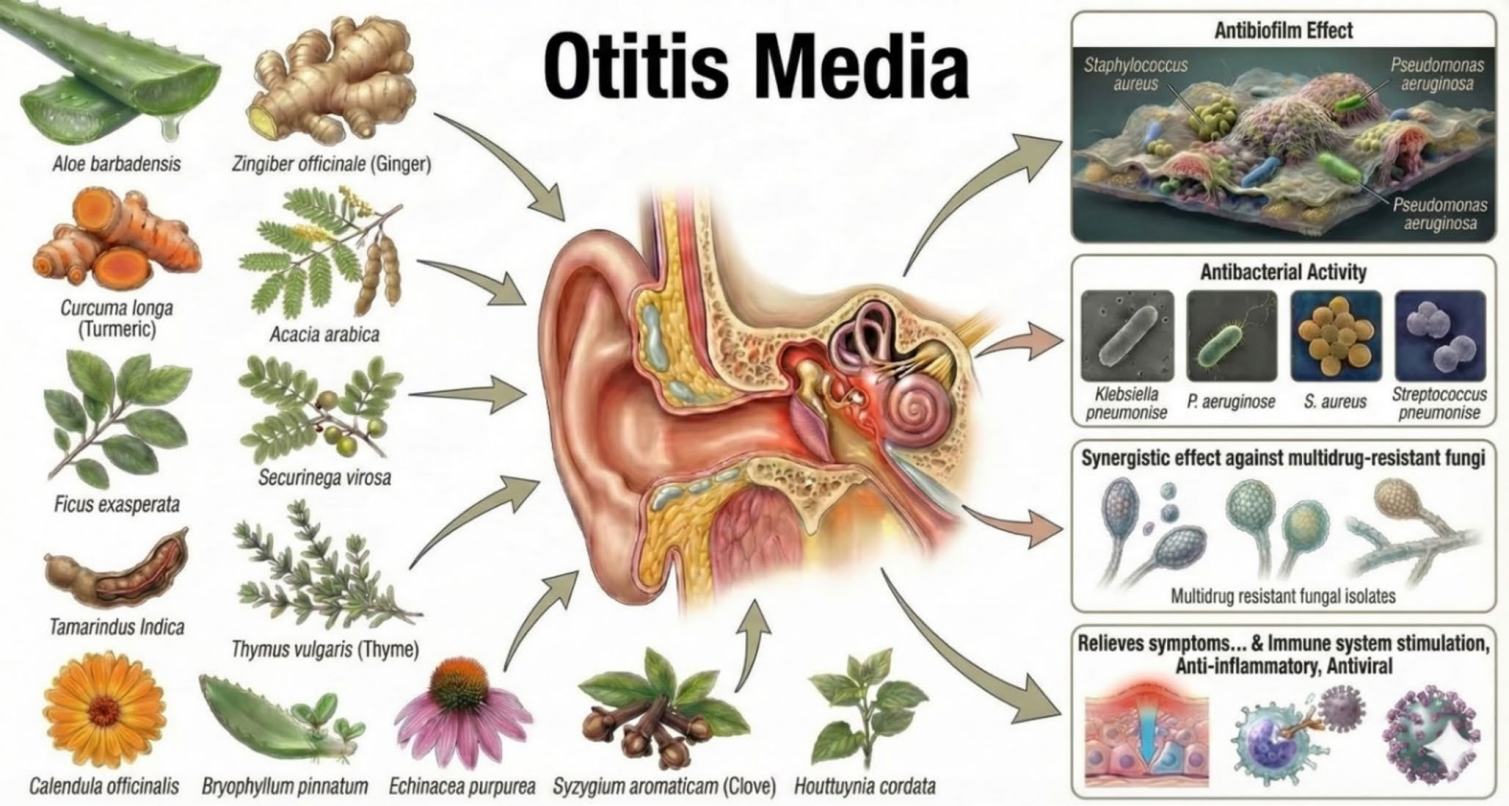
The medicinal plants and their pharmacological activities used for otitis media

*Houttuynia cordata H. cordata* is a traditional medicinal plant particularly used in East Asia to treat upper respiratory tract and lung infections. Lu et al. (2006) evaluated the anti-inflammatory effect of *H. cordata* injection (HCI) in rat pleurisy and mouse ear oedema models, demonstrating that HCI administration significantly reduced exudate volume, protein concentration, C-reactive protein levels and leukocyte infiltration. The anti-inflammatory and immunomodulatory effects of *H. cordata* support its use as a complementary phytotherapeutic agent for upper respiratory tract infections and associated ear pathologies. However, as there are few well-designed clinical or In vitro studies directly addressing otitis media, further experimental and clinical data are required to support its routine use for this condition (Lu et al. 2006) (Fig. 5; Table 3).

**Table 3:** Medicinal plants used for Otitis media

Plant species	Chemical components	Exracts methods	Treatment	Model	Pharmacological effect	Target species	References
*Aloe barbadensis*	Anthraquinones (aloin, emodin), polysaccharides, saponins, vitamins (A, C, E), enzymes	Ethanol extraction	Otitis media	In vitro	Antibiofilm effect	*S.aureus*,* P. aeruginosa*	Jotic et al. (2024)
*Zingiber officinale*	Gingerol, shogaol, zingerone, sesquiterpenes, volatile oils (zingiberene)	Ethanol extraction	Otitis media	In vitro	Antibiofilm effect	*S. aureus*,* P.aeruginosa*	Jotic et al. (2024)
*Curcuma longa*	Curcumin, demethoxycurcumin, bisdemethoxycurcumin, volatile oils (turmerone, zingiberene)	Ethanol extraction	Otitis media	In vitro	Antibiofilm effect	*S.aureus*,* P.aeruginosa*	Jotic et al. (2024)
*Acacia arabica*	Tannins, flavonoids, polysaccharides, saponins	Ethanol extraction	Otitis media	In vitro	Antibiofilm effect	*S.aureus*,* P.aeruginosa*	Jotic et al. (2024)
*Ficus exasperate*	Flavonoids, phenolic compound	Methanol extraction	Otitis media	In vitro	Antibacterial Activity	*K.pneumoniae*,* P.aeruginosa*,* S.aphylococcus aureus*,* S.pneumoniae*	Jotic et al. (2024)
*Securinega virosa*	Alkaloids (securinine), flavonoids, tannins	Methanol extraction	Otitis media	In vitro	Antibacterial Activity	*K. pneumoniae*,* P.aeruginosa*,* S.aureus*,* S.pneumoniae*	Jotic et al. (2024)
*Tamarindus indica*	Polysaccharides, organic acids (tartaric acid), phenolic compounds, flavonoids	Methanol extraction	Otitis media	In vitro	Antibacterial Activity	*K.pneumoniae*,* P.aeruginosa*,* S.aureus*,* S.pneumoniae*	Jotic et al. (2024)
*Thymus vulgaris*	Thymol, carvacrol, linalool, pinene, flavonoids	Essential oil	Otitis media	In vitro	synergistic effect against multidrug-resistant fungi	*Multidrug-resistant fungal isolates*	Ghaly et al. (2020)
*Syzygium aromaticum*	Eugenol, vanillin, methyl eugenol, flavonoids	Extrate	Otitis media	In vitro	synergistic effect against multidrug-resistant fungi	*Multidrug-resistant fungal isolates*	Ghaly et al. (2020)
*Calendula officinalis*	Flavonoids (quercetin, isorhamnetin), triterpenoids, carotenoids, saponins	Water-based extraction	Otitis media	In vitro	relieves symptoms of otitis media		Petran et al. (2024)
*Bryophyllum pinnatum*	Flavonoids, bufadienolide glycosides, alkaloids, phenolic compounds	Water-based extraction	Otitis media	In vivo	Antibacterial Activity	*S. aureus*,* P.aeruginosa*	Sani (2020)
*Echinacea purpurea*	Alkamides, polysaccharides, glycoproteins	Liquid extract, tinctures	Upper respiratory tract infections, immune system support	Immune system stimulation, anti-inflammatory, antimicrobial	Human	PMC4441164, PMC9102300	Taufani and Ha (2025)
*Houttuynia cordata*	Essential oils (methyl methacrylate, methyl eugenol), flavonoids, alkaloids	Steam distillation, alcohol extraction	Antibacterial, antiviral, anti-inflammatory treatments	Immune modulation, antioxidant, antitumor	Human	PMC9501394, PMC7127097	Lu et al. (2006)

### Tinnitus

*Rosa canina*,* Urtica dioica*,* Tanacetum vulgare* Khosravi et al. (2022) was examined the effects of the Neurotec^®^ product, containing a combination of *R. canina*,* U. dioica*, and *T. vulgare*, on tinnitus. The study demonstrated that the combination protected neuronal cells by reducing oxidative stress, suppressed inflammatory cytokine levels, and enhanced nerve conduction. These mechanisms contributed to a marked improvement in tinnitus symptoms.

*Hypericum perforatum* + *Ginkgo biloba* This herbal combination of *H. perforatum* and *G. biloba*, containing hypericin and flavonoids, contributed to the regulation of neurotransmitters such as serotonin, dopamine, and GABA. The study observed enhanced synaptic transmission, balanced neuronal activity, and a reduction in tinnitus perception. Furthermore, the extracts showed antimicrobial activity against *S.aureus* and *E. coli*. The effects of *H. perforatum* extract were investigated on tinnitus in an in vivo study. Due to its hypericin and hyperforin content, the plant plays a role in regulating neurotransmitters such as serotonin and noradrenaline, balancing neuronal transmission and reducing tinnitus severity. It also exhibited antimicrobial effects against *S. aureus* and *Streptococcus spp*. The study reported that the plant both suppressed neuroinflammation and improved synaptic function (Kim et al. 2024).

*Euterpe oleracea* The fruit exract of *E. oleracea* was neutralised free radicals and reduced oxidative stress with its high antioxidant capacity. The study demonstrated reduced neuronal damage, suppressed inflammatory response, and alleviated neural sensitivity associated with tinnitus (Liu et al. 2023).

*Mentha piperita* The essential oils of *M. piperita* has been shown to be effective against oxidative stress and inflammation associated with tinnitus. Due to its menthol and flavonoid composition, *M. piperita* extract demonstrated considerable antioxidant capacity, mitigating cellular deterioration by curbing inflammatory mediators. It has also been shown to have antimicrobial activity against *E. coli* and *S. aureus* (Inoue and Hayashi 2023).

**Fig. 6:**
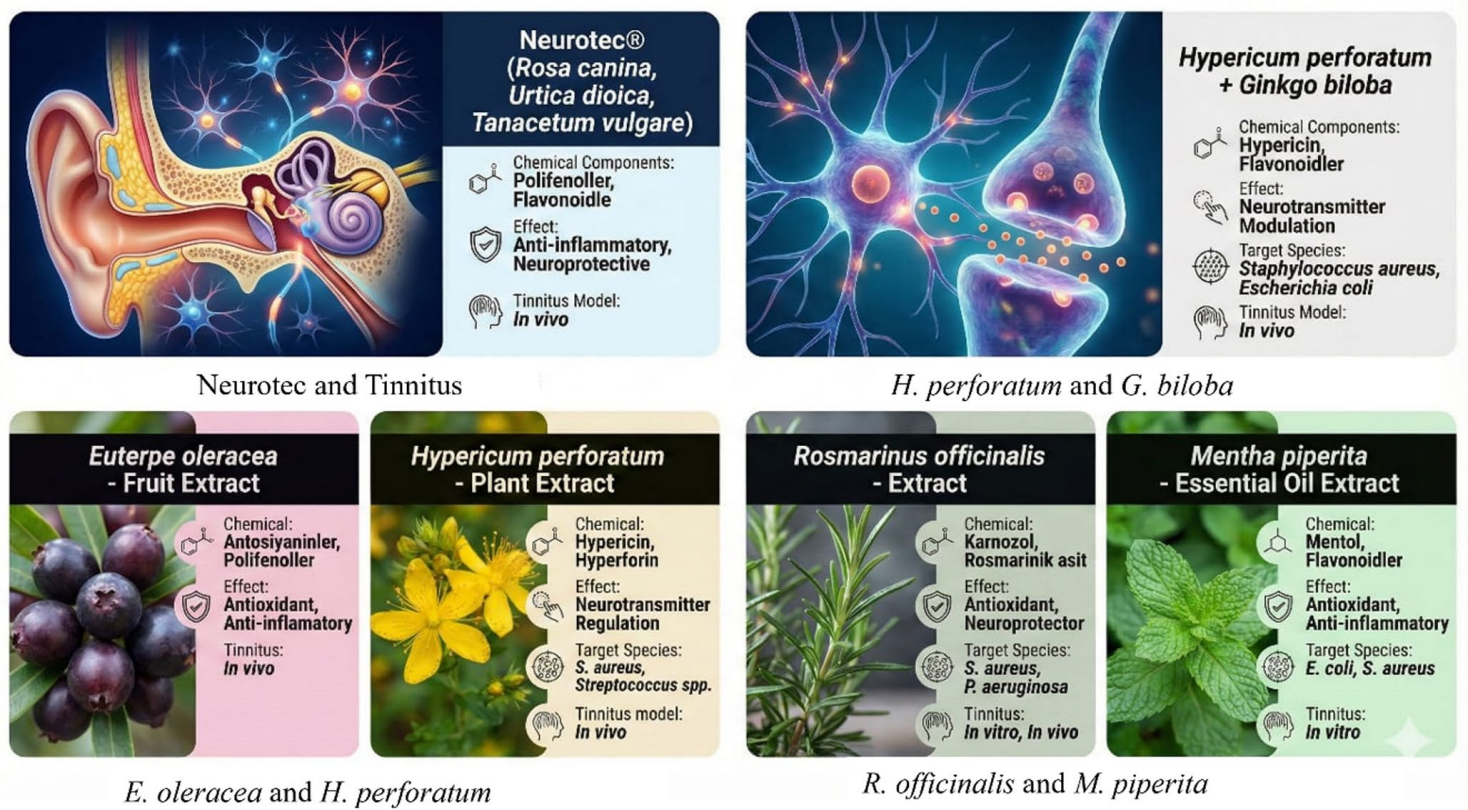
The medicinal plants and their pharmacological activities used for tinnitus

*Rosmarinus officinalis* The *R. officinalis* extract showed potent antioxidant and neuroprotective effects, neutralised free radicals and protected neuronal cells from oxidative damage. It also suppressed inflammation by reducing the release of pro-inflammatory cytokines such as IL-1β and TNF-α, leading to an improvement in tinnitus symptoms. Additionally, it exhibited antimicrobial effects against *S. aureus* and *P. aeruginosa* (Liu et al. 2023) (Fig. 6; Table 4).

**Table 4:** The medicinal plants used for tinnitus

Plant species	Chemical components	Exracts methods	Treatment	Model	Pharmacological effect	Target species	References
*Rosa canina*,* Urtica dioica*,* Tanacetum vulgare*	Polifenols, Flavonoids	Plant combination(Neurotec^®^ kapsül)	Tinnitus	In vivo	Anti-inflammatory, neuroprotective	Human (103 patients)	Khosravi et al. (2023)
*Hypericum peforatum* + *Ginkgo biloba*	Hypericin, Flavonoids	Combined plant extracts	Tinnitus	In vivo	Neurotransmitter modulation	*S. aureus*,* E.coli*	Kim et al. (2024)
*Euterpe oleracea*	Antocyanins, Polifenols	Fruit extract	Tinnitus	In vivo	Antioxidant, anti-inflammatory	Human (30 patients)	Liu et al. (2023)
*Hypericum perforatum*	Hypericin, Hyperforin	plant extract	Tinnitus	In vivo	Neurotransmitter regulation	*S. aureus*,* Streptococcus spp.*	Kim et al. (2024)
*Rosmarinus officinalis*	Karnozol, Rosmarinic csit	Extract	Tinnitus	In vitro and in vivo	Antioxidant, neuroprotector	*S. aureus*,* P. aeruginosa*	Liu et al. (2023)
*Mentha piperita*	Menthol, Flavonoids	Essential oil extract	Tinnitus	In vitro	Antioxidant, anti-inflammatory	*E.coli*,* S. aureus*	Inoue and Hayashi (2023)

### Vertigo

*Ginkgo biloba G. biloba* has been shown to reduce the severity of vertigo by improving cerebral circulation and supporting vestibular function. A randomised, double-blind study showed that it is as effective as betahistine, commonly used in vertigo treatment, and may have a safer profile (Sokolova et al. 2014) Traditional Chinese herbal formulations exert therapeutic effects on vertigo by modulating vestibular function through the synergistic integration of multiple herbal components. Particularly in the treatment of cervical vertigo (neck-related dizziness), its efficacy has been observed to increase when combined with other treatments such as manual therapy and acupuncture (Oh et al. 2022).

*Zingiber officinale Z.officinale* is known for its potent antiemetic (anti-nausea) effects, particularly in reducing nausea associated with vestibular migraine and motion sickness. It exerts this effect on the gastrointestinal system and is not thought to directly affect the vestibular system. Its effectiveness in reducing nausea caused by motion sickness and vestibular migraine has been demonstrated in various clinical studies (Ciuman 2012).

*Melissa officinalis M. officinalis* has anxiolytic and sedative properties that exert sedative effects on the central nervous system. These properties may help manage symptoms such as anxiety and tension associated with vertigo, and it is believed that this relaxation is achieved through its effect on the GABA system. The essential oils of M. officinalis is used particularly to improve residual dizziness following vertigo (Chiarella et al. 2021).

*Equisetum arvense E. arvense* is primarily known for its antihypertensive effect, and a clinical study has demonstrated that it significantly reduces systolic and diastolic blood pressure. Although no study has been established with vertigo, it may provide an indirect benefit in cases where hypertension causes vertigo symptoms (Carneiro et al. 2022) (Table 5).

**Table 5:** Medicinal plant used for vertigo

Plant species	Chemical components	Exracts methods	Treatment	Model	Pharmacological effect	Target species	References
*Ginkgo biloba*	Flavonoids (kaempferol, quercetin), terpenoids (ginkgolide, bilobalide)	Ethanol extraction	Vertigo	In vitro and in vivo	Modulates vestibular functions contributing to alleviation of vertigo symptoms; exhibits antioxidant and neuroprotective effects.	*S.aureus*, *E. coli*	Sokolova et al. (2014)
*Zingber officinale*	Gingerols, shogaols, zingeron	Ethanol or water extraction	Vertigo	In vitro and in vivo	Demonstrated antiemetic effects that reduce vertigo-associated nausea in vestibular migraine and motion sickness.	*Helicobacter pylori*, *Salmonella*	Ciuman (2012)
*Melissa officinalis*	Rosmarinic acid, flavonoids, essential oils	Ethanol and water extraction	Vertigo	In vitro	Exhibits anxiolytic and sedative properties, exerting calming effects on the central nervous system that may aid in managing vertigo-related symptoms.	*Staphylococcus aureus*	Chiarella et al. (2021)
Chinese herbal formulations	Multiple plant compounds (example: ginsenosides, flavonoids)	Dried herbal mixtures	Vertigo	In vivo	Synergistic effects of multiple components contribute to regulation of vestibular function and reduction of vertigo symptoms.	Multiple effects, limited direct effect on bacteria	Oh et al. (2022)
*Equisetum arvense*	Silica, flavonoids (quercetin, kaempferol), phenolic acids, saponins, alkaloids, minerals (K, Ca) — (pharmacognostic reports)	Aqueous decoction or hydroalcoholic (ethanol) extracts; standardized dry extracts used in clinical trials. (see PhcogRev, MDPI reviews).	Equisetum arvense extract (clinical use for hypertension)	Double-blind randomized clinical trial – Humans	Antihypertensive effect (↓ SBP, DBP)	Human	Carneiro et al. (2022)

### Medicinal plants used in nose diseases

#### Alergic rhinitus

*Astragalus membranaceus*, *Glycyrrhiza glabra* The study examined the synergistic effects of *A. membranaceus*, *G. glabra* present in the traditional water-based decoction formulation. According to the findings, this herbal combination exhibited strong anti-inflammatory effects through inhibition of the NF-κB signaling pathway, effectively reduced inflammation via the IFNG/IRF1 axis, and significantly lowered serum IgE levels. The microbial target spectrum included pathogenic microorganisms such as *S. aureus* and *E. coli*. These results suggest that this traditional Chinese medicinal herbal combination may serve as a scientifically supported alternative for modern allergic rhinitis treatment (Deng et al. 2025).

*Petasites hybridus* Ze 339 is prepared from the plant leaves from *P. hybridus* using a specialized extraction method and is administered in standard doses in tablet or capsule form. The observational study included 226 patients, and Ze 339 was found to alleviate symptoms by reducing histamine release and inhibiting mast cell degranulation. Overall, Ze 339 emerges as a safe and effective phytotherapeutic alternative that significantly reduces allergic rhinitis symptoms, demonstrating comparable efficacy and safety to synthetic antihistamines (Merk et al. 2025). In a another study, leaf extracts of *P. hybridus* were comprehensively evaluated for their therapeutic value in allergic rhinitis using ethanol and water extraction methods. Antimicrobial spectrum analysis indicated efficacy against Gram-positive bacteria such as *S. aureus* and *Streptococcus* species. These findings suggest that *P. hybridus* extract may serve as a versatile therapeutic agent for controlling allergic rhinitis symptoms and offer a valuable complementary alternative to conventional treatments (Blosa et al. 2021).

*Allium ascalonicum* In a randomized controlled clinical study the potential of *A. ascalonicum* in the treatment of allergic rhinitis was comprehensively investigated both In vitro using the RBL-2H3 cell line and in a 4-week clinical trial involving 16 patients. The study evaluated the pharmacological activity of quercetin derivatives obtained through daily consumption of approximately 1.5 bulbs (3 g) of fresh shallots, specifically quercetin 3,4’-diglucoside and quercetin 4’-glucoside. The findings indicated that these phytochemicals achieved up to 97% inhibition of the β-hexosaminidase enzyme, demonstrating remarkable efficacy in mast cell stabilization and playing a critical role in suppressing histamine release. This study highlights the significant clinical potential of a natural, diet-integrable approach for managing allergic rhinitis symptoms (Arpornchayanon et al. 2022).

*Magnolia* sp. Advances in phytochemical isolation techniques have facilitated the purification of volatile oils from *M. biondii* demonstrating that cinnamyl ester derivatives effectively suppress histamine release, mitigate inflammatory signaling pathways, and promote immunomodulatory activity. Notably, these constituents reduce the inflammation-induced secretion of the neuropeptide substance P and inhibit type I hypersensitivity responses. Substance P is an 11-amino-acid neuropeptide belonging to the tachykinin family. It is widely distributed in the central and peripheral nervous system and plays a key role in pain perception, inflammation, and neuroimmune signaling. Collectively, this multi-component, multi-target synergistic profile offers a mechanistic rationale for their application in the management of allergic rhinitis (Dong et al. 2025).

*Scutellaria baicalensis* The study explored baicalin’s from the roots of *S. baicalensis* ability to modulate the Th17/Treg cell balance by inhibiting autophagy processes, resulting in decreased pro-inflammatory IL-17 A levels and increased anti-inflammatory IL-10 levels. The findings indicate that baicalin plays a critical role in restoring immune system homeostasis, positioning it as an effective therapeutic agent for controlling allergic inflammation. This research provides important scientific evidence supporting the use of *S. baicalensis* in traditional Chinese medicine from a modern immunological perspective (Xu et al. 2025).

*Perilla frutescens* The study, focused on the roles of the rosmarinic acid, luteolin, and apigenin *from P. frutescens*——in suppressing Th2 immune responses. Results demonstrated that the extract significantly reduced levels of Th2-type cytokines, including IL-4, IL-5, and IL-13, thereby effectively controlling allergic inflammation. Additionally, the extract exhibited antimicrobial activity against pathogenic bacteria such as *E. coli* and *S. aureus*. These findings suggest that *P. frutescens* offers dual therapeutic potential in allergic rhinitis through its combined anti-allergic and antimicrobial properties (Bival Štefan 2024).

*Curcuma longa* In a molecular-level study detailed *C.longa* potent antioxidant properties and its efficacy in suppressing the NF-κB signaling pathway. Findings indicated that *C.longa* plays a critical role in reducing oxidative stress and regulating inflammatory responses. Furthermore, the extract exhibited broad-spectrum antimicrobial activity against both Gram-positive and Gram-negative bacteria, including *S. aureus* and *P. aeruginosa*. These results suggest that curcumin may serve as a versatile therapeutic agent in allergic rhinitis and demonstrate how traditional medicinal applications can be grounded in modern scientific evidence (Jayasinghe et al. 2024).

*Origanum vulgare* In a comprehensive phytochemical study showed that the extract of *O. vulgare* effectively disrupted microbial cell membranes and exhibited antimicrobial activity against a broad spectrum of pathogens, including *S. aureus*,* S. pyogenes*,* E. coli*, and *C. albicans*. These findings suggest that *Origanum* vulgare may serve as a valuable phytotherapeutic option in allergic rhinitis, providing both symptomatic relief and prevention of secondary infections (Nabavizadeh et al. 2021).

*Zingiber officinale* The study investigated the role of *Z. officinale* main bioactive constituentsin enhancing immune responses against environmental stressors. Findings indicated that the ginger extract significantly reduced Th2-type cytokine levels, including IL-4, IL-5, and IL-13, effectively stabilized mast cells, and exhibited strong antioxidant activity. This study provides valuable insights from an environmental health perspective, suggesting that ginger may serve as a protective complementary therapy for allergic rhinitis patients living in areas with high urban air pollution (Park et al. 2025).

*Glycyrrhiza glabra* In a controlled laboratory study the roots of *G. glabra* (licorice) was thoroughly evaluated for its immunomodulatory effects in an OVA-induced allergic rhinitis mouse model. Results demonstrated that *G. glabra* extract effectively balanced adaptive immune responses and provided therapeutic benefits in controlling allergic inflammation by regulating T cell function. These findings indicate that licorice, widely used in traditional medicine, can be assessed in modern allergology through a scientific framework and represents a valuable phytotherapeutic option for restoring immune system homeostasis (Liao et al. 2021).

*Pimpinella anisum* In a study demonstrated that the essential oils of *P. anisum* play a significant role in regulating T cell balance and modulating inflammatory responses. Findings indicated that essential oil effectively contributed to the restoration of immune system homeostasis and demonstrated valuable therapeutic properties in modulating allergic responses. This study suggests that *P. anisum*, commonly used in traditional aromatherapy, can be scientifically assessed for allergic rhinitis treatment and may serve as a natural modulator for balancing T cell-mediated immunity (Liao et al. 2021).

*Camellia sinensis* In a study investigated that the *C. sinensis* (tea) extract play the role of potent in regulating helper T cell (Th1/Th2) balance and reducing inflammatory cytokines. Results demonstrated that the tea extract effectively contributed to the restoration of immune system homeostasis and exhibited valuable therapeutic properties in controlling allergic inflammation through modulation of Th1/Th2 polarization. These findings suggest that regularly consumed tea may play a supportive role in allergic rhinitis management and that a polyphenol-rich diet could be significant in preventing allergic diseases (Liao et al. 2021).

*Matricaria recutita* In a study investigated the clinical implications of the mucosal anti-inflammatory effects of *M. recutita* (chamomile) exract. Findings indicated that the chamomile extract effectively stabilized mast cells and contributed to the improvement of mucociliary clearance mechanisms, demonstrating valuable therapeutic properties. Clinical observations revealed that chamomile exerted restorative effects on the nasal mucosa, significantly alleviating allergic symptoms. This study suggests that chamomile, widely used in traditional phytotherapy, may serve as a safe and effective complementary option for allergic rhinitis and could be integrated into clinical practice (Sah et al. 2022). In a systematic study showed that chamomile extracts are generally well tolerated in in vivo applications, with serious adverse effects being rare. The safety evaluation and adverse event monitoring results suggest that chamomile exhibits a favorable safety profile for long-term use in the management of allergic conditions. This systematic review provides valuable reference information, supporting the safe clinical use of chamomile-based phytotherapeutic products (Ostovar et al. 2025).

*Mentha piperita* In a study demonstrated that M.piperita essential oil effectively inhibited pro-inflammatory cytokines such as TNF-α, IL-1β, and IL-6, and played a critical role in preserving epithelial tight junction proteins ZO-1 and occludin. These findings suggest that peppermint essential oil offers a dual therapeutic value by maintaining nasal epithelial barrier function and controlling allergic inflammation. The study highlights that peppermint oil, commonly used in aromatherapy, can be evaluated as a scientifically grounded approach for the treatment of allergic rhinitis (Park et al. 2022).

*Paeonia* sp. In a comprehensive systematic review indicate that monoterpene glycosides of *Paeonia* exhibit versatile pharmacological activities and hold significant therapeutic potential in controlling allergic inflammation. Evaluation of their pharmacological activities revealed that these compounds may effectively contribute to immune modulation, anti-inflammatory effects, and the regulation of allergic responses. This results showed that *Paeonia* species, traditionally used in Chinese medicine, can be scientifically evaluated for modern phytotherapeutic applications and may offer innovative therapeutic options for the treatment of allergic diseases (Zhu et al. 2025) (Table 6).

**Table 6:** Medicinal plant used for allergic rhinitis

Plant species	Chemical components	Exracts methods	Treatment	Model	Pharmacological effect	Target species	References
*Astragalus membranaceus & Glycyrrhiza glabra*	Quercetin, Astragaloside IV, Glisirizin	Water-based decoction	Allergic rhinitis	In vivo	Inhibition of the NF-κB pathway; reduction of inflammation via the IFNG/IRF1 axis; reduction of IgE levels	*Staphylococcus aureus*, *Escherichia coli*	Deng et al. (2025)
*Petasites hybridus*	Sesquiterpenes, petasin, isopetasin	Leaf extract (Ze 339)	Allergic rhinitis	In vivo	Efficacy and safety demonstrated for treatment of allergic rhinitis through anti-inflammatory mechanisms and reduction of nasal symptoms.	Anti-inflammatory effects, reduction of histamine release	Merk et al. (2025)
*Petasites hybridus*	Petacin, Isoopetacin	Leaf extract, Ethanol or water extraction	Allergic rhinitis	In vivo	Anti-inflammatory, antihistamine, mast cell stabilizer	*Staphylococcus aureus*,* Streptococcus spp.*	Blosa et al. (2021)
*Allium ascalonicum*	Quercetin derivatives	Consumption of fresh salsify (3 g / approx. 1.5 heads per day)	Allergic rhinitis	In vitro (RBL-2H3 cell line) and randomized controlled clinical trial (16 patients, 4 weeks)	β-hexosaminidase inhibition (97%), mast cell stabilization, suppression of histamine release	Human (16 patients)	Arpornchayanon et al. (2022)
*Magnolia biondii . (Xinyi)*	1,8-Cineole, β-Cubebene, Trans-Farnesol, Caryophyllene, α-Cadinol, Linalool, Sabinene, β-Pinene, γ-Muurolene		allergic rhinitis	In vivo	Improves trace elements in the body, regulates immune function, and improves nasal ventilation	Human	Dong et al. (2025)
*Scutellaria baicalensis*	Baicalin (flavonoid)	Extraction from plant roots	Allergic rhinitis	In vivo and In vitro	Inhibits autophagy by regulating Th17/Treg balance; decreases IL-17 A, increases IL-10	Th17/Treg cell	Xu et al. (2025)
*Perilla frutescens*	Rosmarinic acid, luteolin, apigenin	Ethanol extraction	Allergic rhinitis	In vivo and In vitro	Suppression of Th2 response, decreased levels of IL-4, IL-5, IL-13	*E. coli*, *S. aureus*	Bival Štefan (2024)
*Curcuma longa*	Curcumin	Ethanol extraction	Allergic rhinitis	In vivo and In vitro	Antioxidant, NF-kB suppression	*S. aureus*,* P.aeruginosa*	Jayasinghe et al. (2024)
*Origanum vulgare*	Carvacrol, thymol, flavonoids, phenolic acids	Leaves and essential oils obtained by distillation or ethanol extraction	Allergic rhinitis	In vivo and In vitro	Anti-inflammatory, antihistamine, mast cell stabilization, immune modulation, microorganism cell membrane disruption	*S. aureus*,* S. pyogenes*,* E. coli*,* C.albicans*	Nabavizadeh et al. (2021)
*Zingiber officinale Roscoe*	Gingerol, shogaol, zingeron, paradol	Ethanol extraction from the root	Allergic rhinitis	Animal model of allergic rhinitis, exacerbated by particulate matter (air pollution)	Reduction of cytokine levels (IL-4, IL-5, IL-13), mast cell stabilization, antioxidant effects	Mast cell	Park et al. (2025)
*Glycyrrhiza glabra*	Glycyrrhizin, flavonoids	Ethanol extraction from the root	Allergic rhinitis	Mouse model of allergic rhinitis induced by OVA, laboratory environment	Modulation of T cell activation	T cell	Liao et al. (2021)
*Pimpinella anisum*	Anethol, flavonoids	Essential oil extraction from seed	Allergic rhinitis	Mouse model of allergic rhinitis induced by OVA, laboratory environment	T cell balance and inflammation regulation	T cell	Liao et al. (2021)
*Camellia sinensis*	Catechins, polyphenols	Hot water extraction from leaves	Allergic rhinitis	Mouse model of allergic rhinitis induced by OVA, laboratory environment	Helper T cell (Th1/Th2) balance, decreased inflammatory cytokines	T cell	Liao et al. (2021)
*Matricaria recutita*	Apigenin, bisabolol, flavonoids, coumarin derivatives	water extraction	Allergic rhinitis	Clinical study (human subjects)	Mucosal anti-inflammatory effect, mast cell stabilization, increased mucociliary clearance	Human	Sah et al. (2022)
	Chamazulene, bisabolol, apigenin	Systematic extraction review	Allergic conditions	In vivo	Systematic review of adverse events and safety profile of chamomile in allergic conditions.	Safety assessment, adverse event monitoring	Ostovar et al. (2025)
*Mentha piperita*	Menthol, menthone, limonene, 1,8-cineole, pulegon	Essential oil	Allergic rhinitis	In vivo - mouse model of allergic rhinitis induced by OVA (ovalbumin)	Reduction of inflammation (inhibition of TNF-α, IL-1β, IL-6) ,Protection of epithelial tight junction proteins (ZO-1, occludin)		Park et al. (2022)
*Paeonia species*	Monoterpene glycosides	Glycoside extraction	Allergic conditions	In vivo	Systematic review on pharmacological activities and molecular mechanisms of Paeonia monoterpene glycosides.	Pharmacological activity assessment	Zhu et al. (2025)

### Rhinosinusitis

*Pelargonium sidoides* The extract of *P. sidoides*, reduces inflammation by lowering chemokine levels in the nasal secretions of patients with acute rhinosinusitis. The chemical constituents of *Pelargonium sidoides* exert their therapeutic effects primarily through immunomodulatory mechanisms. It is effective against bacteria that cause rhinosinusitis, such as *S. pneumoniae* and *H. influenzae* (Perić et al. 2020).

*Thymus vulgaris* The essential of *T. vulgaris* has been shown to modulate pro-inflammatory cytokines in respiratory tract infections. It is effective against rhinovirus, respiratory syncytial virus, and influenza virus, and is used in the treatment of rhinosinusitis (Nocerino et al. 2025).

*Origanum vulgare* A randomised, double-blind study has demonstrated that a nasal spray containing *O. vulgare* essential oil provides clinically significant benefits in patients with chronic rhinosinusitis (CRS) compared to fluticasone and sesame oil. This plant, reduces mucosal inflammation and improves symptoms through its therapeutic effects (Qaraaty et al. 2020).

**Table 7:** Medicinal plant used for rhinosinusitis

Plant species	Chemical components	Exracts methods	Treatment	Model	Pharmacological effect	Target species	References
*Pelargonium sidoides*	Polyphenols, phenolic compounds, coumarin derivatives	Aqueous and ethanolic extraction from the roots of the plant	Rhinosinusitis	In vivo	Reduces inflammation by lowering chemokine levels in nasal secretions; provides immune modulation	*S. pneumoniae*,* H. influenzae*,* M. catarrhalis*	Perić et al. (2020)
*Thymus vulgaris*	Thymol, carvacrol, flavonoids	Essential oil and plant extract	Rhinosinusitis	In vivo	Antimicrobial, anti-inflammatory, mucolytic effects	Rhinovirus, RSV, Influenza	Qaraaty et al. (2020)
*Origanum vulgare*	Carvacrol, thymol, p-cymene, γ-terpinene	Essential oil	Rhinosinusitis	In vivo	Anti-inflammatory, antimicrobial, antioxidant effects; reduction of mucosal inflammation and symptom improvement	*S. aureus*,* S.pneumoniae*,* H.influenzae*	Tiboc Schnell et al. (2021)
*Sambucus nigra*	Anthocyanins, flavonoids, polyphenol	Aqueous extract	Rhinosinusitis	In vivo	Anti-inflammatory, antioxidant, tissue remodeling regulator	*Escherichia coli* LPS-induced inflammation model	Tiboc Schnell et al. (2021)
*Matricaria aurea*	Flavonoids (apigenin, luteolin, quercetin, patuletin), α-bisabolol, chamazulene	Dry extract, essential oil	Rhinosinusitis	In vitro, in vivo	Anti-inflammatory, antimicrobial, antispasmodic	*B.subtilis*, *S. pyogenes*, *S.aureus*	Alkheder et al. (2024)
*Matricaria recutita*	Chamazulen, farnesene, flavonoids (apigenin, quercetin	Dry extract, essential oil	Rhinosinusitis	In vitro, in vivo	Anti-inflammatory, antiseptic, wound healing	*B.subtilis*, *S. pyogenes*, *S.aureus*	Alkheder et al. 2024
*Curcuma longa*	Curcumin	In silico analysis	Rhinosinusitis	In silico	Binding to HRV14 capsid protein	Human Rhinovirus 14 (HRV14)	Dash et al. (2024)
*Allium sativum*	Allicin, flavonoids	Garlic skin extract ethnol extract	Rhinosinusitis	In vivo	Reduction of NO (Nitric Oxide) and TNF-α levels in acute bacterial sinusitis	*S. aureus*,* S. pneumoniae*	Wijayanti et al. (2024)

*Sambucus nigra* The intranasal application of *S. nigra* extract has been investigated for its beneficial effects on inflammation, oxidative stress, and tissue remodelling in a subacute rhinosinusitis rat model induced by lipopolysaccharide (LPS). This plant, rich in anthocyanins, flavonoids, and polyphenols, has demonstrated activity in an *E. coli* LPS induced inflammation model and may therefore represent a promising therapeutic option for the treatment of rhinosinusitis (Tiboc Schnell et al. 2021)

*Matricaria aurea & Matricaria recutita* A nasal drop containing chamomile extract has been shown to further reduce clinical symptoms and improve quality of life in patients with chronic rhinosinusitis. These plants are known for their anti-inflammatory, antimicrobial and antispasmodic effects (Alkheder et al. 2024).

*Curcuma longa* In silico studies have indicated that curcumin, the active component of *C. longa*, may play a role in viral upper respiratory tract infections due to its ability to bind to the capsid protein of Human Rhinovirus 14 (HRV14), which causes the common cold (Dash et al. 2024).

*Allium sativum A.sativum* peel extract has been shown to reduce nitric oxide (NO) and TNF-α levels in rats with acute bacterial sinusitis. Garlic, may be beneficial in the treatment of acute bacterial sinusitis due to its anti-inflammatory effect (Wijayanti et al. 2024) (Table 7).

### Medicinal plants used in throat diseases

#### Pharyngitis

*Salvia officinalis* In a study demonstrated that *S.officinalis* essential oil effectively in vivo model of post-operative hoarseness. These findings suggest that *S.officinalis* essential oil exhibited notable sedative and anti-inflammatory properties and demonstrated activity against *S.pyogenes* and *S. aureus*. The study highlights that S.officinalis oil, commonly used in aromatherapy, can be evaluated as a scientifically grounded approach for the treatment of pharyngitis (Katebian et al. 2023)

*Althaea officinalis* In a studies, A.officinalis extractwas tested in both in vivo and clinical studies for the treatment of pharyngitis, throat irritation, and cough. It demonstrated mucosal protective, anti-inflammatory, and soothing effects, and was reported to be effective against *S. mutans* and *H.influenzae* (Kręgielczak et al. 2023).

*Glycyrrhiza glabra* Both aqueous and ethanolic extracts of *G. glabra* were evaluated in in vivo and clinical models for the treatment of pharyngitis, hoarseness, and throat inflammation. It demonstrated anti-inflammatory and antimicrobial effects, provided protective effects on the mucosa, and was found to be effective against *S. pyogenes* and *E. coli*. This study suggests that *G. glabra*, widely used in traditional phytotherapy, may serve as a safe and effective complementary option for pharyngitis and could be integrated into clinical practice (Armanini et al. 2020)

*Matricaria chamomilla* The extract of *M. chamomilla* was tested in both in vivo and In vitro models for the treatment of pharyngitis, mucosal irritation, and inflammation. The extracts demonstrated anti-inflammatory, antioxidant, and mucosa-soothing properties, and were found to be effective against *S. aureus* and various *Streptococcus* species (Srivastava and Gupta 2015).

*Thymus vulgaris* In a study, essential oil and extract of *T. vulgaris* were found to be effective in the treatment of pharyngitis and bacterial throat infections in both In vitro and clinical models. The study demonstrated notable antimicrobial, antiseptic, and anti-inflammatory properties, and was shown to be effective against *S. pyogenes* and *H. influenzae* (Kostić et al. 2020) (Table 8).

**Table 8:** Medicinal plant used for Pharyngitis

Plant species	Chemical components	Exracts methods	Treatment	Model	Pharmacological effect	Target species	References
*Salvia officinalis*	Rosmarinic acid, carnosol, essential oils (thujone, cineole)	Ethanolic extract, essential oil	Pharyngitis, sore throat, inflammation	In vivo and clinical studies	Anti-inflammatory, antimicrobial, soothing effect on throat mucosa	*S. pyogenes*,* S. aureus*	Katebian et al. (2023)
*Althaea officinalis*	Mucilage polysaccharides, flavonoids, phenolic acids	Aqueous extract, syrup	Pharyngitis, throat irritation, cough relief	In vivo and clinical studies	Mucosal protective, anti-inflammatory, soothing	*S. mutans*,* H. influenzae*	Kręgielczak et al. (2023)
*Glycyrrhiza glabra*	Glycyrrhizin, liquiritigenin, flavonoids	Water or ethanol extract	Pharyngitis, hoarseness, throat inflammation	In vivo and clinical studies	Anti-inflammatory, antimicrobial, mucosal protection	*S. pyogenes*,* E.coli*	Armanini et al. (2020)
*Matricaria chamomilla*	Apigenin, chamazulene, bisabolol, flavonoids	Infusion, ethanol extract	Pharyngitis, mucosal irritation, inflammation	In vivo and In vitro	Anti-inflammatory, antioxidant, soothing mucosa	*S. aureus*,* S.species*	Srivastava and Gupta (2015)
*Thymus vulgaris*	Thymol, carvacrol, flavonoids, tannins	Essential oil, infusion	Pharyngitis, bacterial throat infections	In vitro and clinical studies	Antimicrobial, antiseptic, anti-inflammatory	*S. pyogenes*,* H.influenzae*	Kostić et al. (2020)

### Cough

*Glycyrrhiza glabra* Ghaemi et al. (2020) conducted a study investigating the effect of *G. glabra* (liquorice root) in lozenge form on chronic cough with 70 patients. The extract have been shown to reduce cough severity by inhibiting the cough reflex and reducing bronchial irritation. Furthermore, the antioxidant and anti-inflammatory properties of liquorice root extract reduce inflammation in the respiratory tract and promote mucosal healing. The study revealed that liquorice sweets could be considered as an alternative treatment for chronic cough.

*Thymus vulgaris* In a study showed that syrup from essential oil of *T. vulgaris* reduce cough severity by inhibiting the cough reflex and reducing bronchial irritation. Furthermore, thyme components suppress the NF-κB and COX-2 pathways, reducing the release of pro-inflammatory cytokines (IL-1β, TNF-α, IL-6) and decreasing oedema and inflammation in the bronchial mucosa. The study shows that thyme syrup may be an effective and safe alternative in cough treatment through both antitussive and anti-inflammatory mechanisms (Eskandarpour et al. 2024).

*Eucalyptus globulus* The essential oil of *E. globulus* reduce the severity of cough by decreasing inflammation in the bronchial mucosa and regulating mucus production. Furthermore, anti-inflammatory effects occur through the suppression of pro-inflammatory cytokines (IL-1β, TNF-α) and modulation of NF-κB pathways. The antimicrobial effects of the essential oil contribute to the treatment of respiratory tract infections by inhibiting pathogens such as *S. pneumoniae*,* H. influenzae*, and *S. aureus* (Öztürk and Safçi 2022).

*Mentha piperita* In a study, the effect of the essential oil of *M. piperita* (peppermint) on coughing was investigated. The severity of coughs is reduced by these essential oils, which inhibit the cough reflex and reduce bronchial irritation. Furthermore, the anti-inflammatory effects of peppermint essential oil were reduced inflammation in the respiratory tract and decrease mucosal oedema by reducing PGE2 and nitric oxide (NO) production (Al-Mijalli et al. 2022) (Table 9).

**Table 9:** Medicinal plant used for cough

Plant species	Chemical components	Exracts methods	Treatment	Model	Pharmacological effect	Target species	References
*Glycyrrhiza glabra*	Glycyrrhizin, liquiritin, isoliquiritigeni	Licorice root extract	Cough	In vivo	Antitussive effect, Reducing bronchial irritation, Antioxidant and anti-inflammatory effect	Human (35 patients)	Ghaemi et al. (2020)
*Thymus vulgaris*	Thymol, carvacrol, flavonoids	Cold extraction with water/ethanol mixture	Cough	In vivo	Antitussive effect, Reducing bronchial irritation, Antioxidant and anti-inflammatory effect	*S. aureus*,* S.pneumoniae*	Eskandarpour et al. (2024)
*Eucalyptus globulus*	1,8-cineole (eucalyptol), α-terpineole, limonene, pinene, flavonoids, tannins	Essential oils are usually extracted by steam distillation	Cough	In vivo	anti-inflammatory and mucolytic effects on the respiratory tract, antimicrobial activity	*S. pneumoniae*,* H. influenzae*,* S. aureus*	Öztürk and Safçi (2022)
*Mentha piperita*	Menthol, menthone, pulegon	Hydrodistillation	Cough	In vivo	Anti-inflammatory effect in carrageenan-induced edema model; reduction of PGE2 and NO production	*S.aureus L. monocytogenes B. subtilis E.coli *,* S. typhimurium*,* P.aeruginosa*	Al-Mijalli et al. (2022)

### Hoarseness

*Rosmarinus officinalis* In a study conducted, the effect of R. officinalis (rosemary) was investigated on post-intubation hoarseness and throat irritation in vivowith 116 patients. The essential oils of *R. officinalis* significantly reduced the expression of pro-inflammatory cytokines, including IL-1β and TNF-α, in the laryngeal mucosa, while also attenuating free radical induced oxidative stress. These mechanisms were reduced of oedema and inflammation in the vocal cords, thereby improving voice quality and functional recovery. Furthermore, In vitro studies have demonstrated that rosemary extract exhibits antibacterial activity against pathogens such as *S. aureus*,* E. coli*, and *P. aeruginosa*. In clinical applications, side effects are minimal, and the treatment has been found to be safe (Safavi et al. 2024).

*Lavandula angustifolia* It has also been reported that L. angustifoliaextract exhibits antimicrobial activity against pathogens such as *S. pyogenes* and *S. aureus* and modulating histamine, prostaglandin, and NF-κB pathways in the laryngeal mucosa and suppressing the inflammatory response. The development of oedema and irritation in the vocal cords is reduced by these mechanisms, and voice quality and functional performance after surgery are significantly improved. In clinical applications, side effects were observed to be minimal and temporary, and the treatment was found to be safe (Dehghan et al. 2025).

*Glycyrrhiza glabra* The effects of *G. glabra* (liquorice root) extract were investigated on hoarseness and laryngeal inflammation. The liquorice root extract were significantly reduced of oedema and inflammation in the vocal cords. The participants reported an improvement in voice quality and they were speaking for longer. No side effects were observed, or they were very mild, indicating that liquorice root is a safe treatment option (Karimi et al. 2022).

*Camellia sinensis* In a study, the effect of *C. sinensis* (green tea) was investigated on throat irritation and voice disorders. A total of 102 patients were included in the study, and participants were randomly assigned to three groups The results showed that, extracts were reduced oedema and irritation in the vocal cords and provided a significant improvement in voice quality. It also exhibited antimicrobial activity against pathogens such as *S. aureus* and *S.mutans*. Side effects were observed at minimal levels, and the study revealed that it is promising as a phytotherapeutic adjuvant in the treatment of hoarseness and throat irritation (Karimi et al. 2022) In a study conducted by Chung et al. 2020, a glycerine-containing spray formulation of the *C. sinensis* was investigated in patients with functional voice disorders. The formulations were reduced inflammation in the vocal cords and demonstrated protective effects on the mucosa in in vivo applications. The study observed a marked improvement in hoarseness and throat irritation following spray application. Side effects were minimal, and the application was found to be safe (Chung and Youn 2020).

*Thymus vulgaris* In a study, the pastille form of the *T. vulgaris* (thyme) was investigated in vivo for the treatment of post-infectious hoarseness and acute pharyngitis. The study observed that thyme pastills significantly reduced symptoms of hoarseness and sore throat. Side effects were minimal, and the treatment was found to be safe (Waheed et al. 2024) (Table 10).

**Table 10:** Medicinal plant used for hoarseness

Plant species	Chemical components	Exracts methods	Treatment	Model	Pharmacological effect	Target species	References
*Rosmarinus officinalis*	Rosmarinic acid, carnosic acid	Essential oil	Hoarseness due to tracheal intubation	In vivo	Anti-inflammatory, antioxidant	*S. aureus*, *E. coli*, *P. aeruginosa*	Dehghan et al. (2025; Safavi et al. (2024)
*Lavandula angustifolia*	Linalool, linalil acetate	Essential oil	Post-operative hoarseness	In vivo	Sedative, anti-inflammatory	*S.pyogenes*, *S. aureus*	Safavi et al. (2024)
*Glycyrrhiza glabra*	Glycyrrhizin, lyciricin	Water extract	Hoarseness, cough	In vivo	Anti-inflammatory, mucosal protection	*H. influenzae*, *S. aureus*, *E. coli*	Dehghan et al. (2025)
*Camellia sinensis*	EGCG, catechins	Infusion	Throat irritation and voice disorders	In vivo	Antioxidant, inflammation suppressant	*S. aureus*, *S. mutans*	Karimi et al. (2022)
*Alchemilla vulgaris*	Tannins, flavonoids	Spray in glycerin	Functional voice disorders	In vivo	Anti-inflammatory, mucosal protective	Human	Chung and Youn (2020)
*Thymus vulgaris*	Timol, carvacrol	Pastille	Post-infectious hoarseness, acute pharyngitis	In vivo	Anti-inflammatory, antiseptic	Human	Waheed et al. (2024)

### Nanotechnological applications of medicinal plants in ENT disorders

Medicinal plants treatment has been used widely for many years and generally has complex formulations, with multiple active components. Unfortunately, many of these active compounds have relatively low bioavailability and solubility, which limits the penetration into their target tissue. Plant materials in traditional herbal medicine are generally of poor bioavailability and may be poorly absorbed by the digestive system which creates less therapeutic effects. In traditional herbal preparations, the biological availability of plant ingredients are usually low with poorly absorption in the gastrointestinal tract, which limits therapeutic efficacy (Bolgen et al. 2025; Lijie Wang et al. 2023) Nanotechnological drug delivery systems offer a promising solution to overcome the limitations of conventional herbal therapies. The use of nanoparticles and other nanocarriers can enhance the solubility and bioavailability of herbal active ingredients (Tajne et al. 2025) Currently available are also nanocarriers that enhance the stability of bioactive compounds and their controlled release, for sustained delivery rate of the drug. Such characteristics can be utilized to protect active herbal constituents from premature degradation and deliver specifically at a site of interest, thus providing enhanced treatment (Yang et al. 2025).

In otorhinolaryngological applications the nanotechnology has special advantages by increasing rapid and high performance delivery of herbal actives to target tissues including, nasal mucosa, oral cavity and upper air way epithelia. The nanocarrier based systems undoubtedly increase residence time on the mucosal surface due to sustained as well as controlled release and produce a greater local concentration of drug which ultimately leads to better therapeutic effects. In the treatment of ear, nose and throat disorders, nanotechnology based drug delivery systems help to overcome problems including poor solubility, low permeability and rapid clearance observed in traditional herbal preparations, thus enhancing drug absorption and ultimately treatment efficiency (Clementino et al. 2021; Kathole et al. 2025; Parvin et al. 2025).

In 2024, a work explored using mucoadhesive chitosan nanoparticles to deliver quercetin intranasal application for the management of allergic rhinitis. The positively charged quercetin loaded chitosan nanoparticles showed high drug encapsulation and stability, and they significantly relieved allergic symptoms in an animal model. Treated mice had significantly lower numbers of sneezes and nose rubbings, and reduced levels of IgE and inflammatory cytokines in the nose tissue were found. Strikingly, the nanoparticle formulation was superior to free quercetin as it largely restored cytokine levels and tissue histology close to those in baseline control, while only a slight promotion of recovery was achieved with free quercetin. This indicated that the nanocarrier significantly enhanced the solubility, mucosal retention and antiallergic effect of quercetin in pathological nasal cavity (Mu et al. 2024).

Several herbal medicine formulations designed using nanotechnology have progressed to the clinical trial stage, demonstrating their potential for translation. These formulations have been reported to provide enhanced therapeutic efficacy in several pathologies, including anti-inflammatory, antioxidant and antimicrobial activities. These properties are of interest in the management of diseases affecting the ear, nose and throat (Clementino et al. 2021; Jalili et al. 2023; Kathole et al. 2025). Current research robustly supports the advantages of nanotechnology based drug delivery systems for herbal medicines in otorhinolaryngological applications, confirming improved mucosal targeting, sustained release and enhanced therapeutic outcomes.

Tanshinone IIA is a lipophilic diterpenoid derived from the root of *Salvia miltiorrhiza*, a common herbal medicine. The hybrid nanoparticles loaded with Tanshinone IIA was developed for the treatment of bacterial otitis media achieved. This nanocomposite was composed of a core of silver nanoparticle, a shell chitosan/lysozyme and Tanshinone IIA as external layer. The nanocarrier showed better antimicrobial effect than untreated group at therapeutic level equivalent to ofloxacin infusion in guinea pig middle-ear infection induced by Staphylococcus aureus. The developed formulation when incorporated in biodegradable in situ gel for otic administration sustained the effect with a reduced dosing frequency which can be interpreted as increased residence time and drug release inside middle ear canal (Yu et al. 2020).

In another study in 2025, researchers formulated a new polyherbal phytosome gel for treatment of pharyngitis. Three plant extracts *Acalypha indica*, *Pergularia daemia* and *Coleus amboinicus* with anti-inflammatory and antimicrobial activities were used to prepare phospholipid based phytosome nanoparticles. These nanovesicles were then incorporated into a topical throat gel to achieve better local bioavailability and adhesion with inflamed pharyngeal tissue. The phytosomal herbal gel exhibited favorable pharmaceutical properties and demonstrated strong In vitro efficacy, significantly reducing inflammatory markers in a sore throat model while showing potent antimicrobial activity against bacteria associated with pharyngitis. With better drug encapsulation and targeting delivery in the throat, the herbal nanocarrier is assumed to have prolonged mucosa-residence time and increased efficacy for relieving pharyngitis (Vijayakumar et al. 2025).

While the integration of nanotechnology with herbal medicine offers significant therapeutic potential, several safety concerns remain. In particular, issues related to biocompatibility, cytotoxicity, and long-term toxicity of nano-formulations require careful evaluation. Previous studies have reported that certain nanoparticles may induce oxidative stress, membrane damage, and inflammatory responses depending on their size, surface characteristics, and concentration. Therefore, the establishment of standardized toxicity assessment guidelines, including dose dependent safety profiles and long-term in vivo studies, is essential for their safe clinical application. In addition, regulatory challenges, large-scale production costs, and quality control issues remain significant barriers to the widespread adoption of herbal nanomedicine in clinical practice (Tajne et al. 2025).

### Side effects of medicinal plants used for ENT diseases

The use of medicinal plants in treatment offers significant advantages due to centuries of accumulated empirical knowledge and their complex structures. Many medicinal plants contain phytochemicals with antioxidant, anti-inflammatory, antimicrobial, and immunomodulatory effects, which can be beneficial in the prevention and supportive treatment of chronic diseases. Furthermore, their natural origin and cultural acceptance can increase patient compliance. However, herbal treatments also have some disadvantages. Active ingredient content can vary depending on the plant species, growing conditions, harvest time, and extraction method, making it difficult to determine standard dosages. Incorrect use, high doses, or drug-plant interactions can lead to serious toxic effects; moreover, the uncontrolled use of practices with limited scientific evidence carries the risk of delaying treatment. Therefore, the use of plants in treatment is considered the safest and most effective approach when it is supported by modern scientific data, quality and safety standards are ensured, and it is carried out under the supervision of healthcare professionals. Every herbal product used, especially at high doses or with prolonged use, may have specific side effects and interactions (Ekor 2014; Heinrich and Gibbons 2001).

*S. officinalis* is considered safe at normal doses. However, high doses or excessive use of concentrated essential oil may cause vomiting, excessive salivation, tachycardia, dizziness and convulsions. Furthermore, due to the thujone and thujin compounds present in sage, it is not recommended during pregnancy and breastfeeding (Hubbert et al. 2006). *Thymus vulgaris* is generally does not cause side effects when taken at recommended doses. However, it has been reported that when taken orally as pure thyme oil, it can cause serious effects such as dizziness, vomiting and breathing difficulties. Skin sensitivity may develop with topical applications (Kıroğlu et al. 2022). *Echinacea species* is generally well tolerated with short-term use. The most common side effects are gastrointestinal disturbances (nausea, abdominal pain); allergic skin rashes and occasionally headaches have also been reported. There is a risk of serious allergic reactions in individuals allergic to chamomile or the pine family. There are conflicting data regarding its potential to affect liver enzymes or interact with immunosuppressive medications (David and Cunningham 2019). *Glycyrrhiza glabra* consist the glycyrrhizin components that exhibit mineralocorticoid-like effects. Excessive intake may lead to hypokalaemia (low blood potassium), peripheral oedema, hypertension, and cardiac arrhythmias. Long-term or high-dose use may cause a condition known as pseudoaldosteronism. Caution should be exercised when used in combination with potassium-depleting (diuretic) medications (Fiore et al. 2008).

Although up to 4 g of *Zingiber officinale* per day is considered safe, higher doses may increase reflux, diarrhoea and heartburn. Ginger may increase the effect of blood-thinning medicines (e.g. warfarin) and increase the risk of bleeding. In rare cases, a drop in blood pressure and arrhythmia have been reported (Kıroğlu et al. 2022). When consumed in high doses, *Allium satvum* is known to cause stomach burning, nausea and flatulence. However, it is generally well tolerated. Garlic oils applied topically to the ear canal may cause skin irritation and even chemical reactions. Furthermore, garlic oil should not be used in cases of a perforated eardrum (hole in the eardrum). Garlic may increase the risk of bleeding due to its blood-thinning effect (Rouf et al. 2020). Although studies of *Eucalptus globulus* is safe when inhaled, pure eucalyptus oil can be toxic in young children and at high concentrations. In children, 2–3 mL of pure oil can cause mild CNS depression (drowsiness, dizziness); intake of ≥ 5 mL can be effective to the extent of causing coma. There is a risk of irritation and bronchospasm in the upper respiratory tract. The safety of high doses during pregnancy and breastfeeding is unknown (Agency 2024a). Although studies of *Pelargonium sidoides* is report that it improves sinusitis symptoms, hepatotoxicity has rarely been reported. Long-term high-dose use may increase the risk of liver damage. Liver function should be monitored regularly (Agency 2024b) *Urtica dioica* is generally safe for short-term use. The most commonly reported side effects are sweating, mild stomach upset and skin reactions. In individuals with allergies, hives or respiratory reactions may occur. There may be interactions in individuals with diabetes, hypertension or those taking blood-thinning medication (the plant may affect blood sugar and blood pressure) (Agency 2024c). The ripe berries of *Sambucus nigra* are considered safe when processed correctly. However, due to cyanide precursor compounds found in raw or unripe berries, leaves and stems, nausea, vomiting and diarrhoea may occur. Therefore, fresh elderberry should be avoided except in industrial products. Allergic reactions are rarely seen (National Center for Complementary and Integrative Health 2020).

Mild gastrointestinal side effects (headache, indigestion) of *Petasites hybridus* are commonly reported with standardised pure products for allergic rhinitis. However, pyrrolizidine alkaloids (PAs) naturally occurring in the plant may cause liver toxicity; therefore, registered PA-free preparations should be preferred. Safety during pregnancy and liver disease has not been established (Mihajilov-Krstev et al. 2020).

## Conclusions

Over the past five years, there has been a significant increase in the use of herbal remedies for ear, nose and throat (ENT) conditions worldwide. Many patients prefer herbal treatments to avoid the side effects that antibiotics and other conventional medicines may cause. The global use of herbal remedies for ENT disorders has risen markedly, influenced by perceptions of safety, cultural traditions, accessibility, and economic factors. Although these products are widely preferred for managing common symptoms such as sore throat, cough, and nasal congestion, their clinical efficacy and safety profiles are not always supported by robust evidence. Variability in usage patterns across regions further highlights the need for standardized evaluation. To ensure safe and effective integration into clinical practice, herbal therapies should be guided by scientific validation and professional supervision. However, the limited scientific evidence regarding the efficacy and safety of herbal treatments also carries risks such as misuse and drug-herb interactions. Therefore, it is recommended that herbal treatments for ENT diseases be used under the guidance of a physician and pharmacist, based on scientific data.

## Data Availability

No datasets were generated or analysed during the current study.
